# A genetic map of the chromatin regulators to drug response in cancer cells

**DOI:** 10.1186/s12967-022-03651-w

**Published:** 2022-09-30

**Authors:** Bo Chen, Pengfei Li, Mingyue Liu, Kaidong Liu, Min Zou, Yiding Geng, Shuping Zhuang, Huanhuan Xu, Linzhu Wang, Tingting Chen, Yawei Li, Zhangxiang Zhao, Lishuang Qi, Yunyan Gu

**Affiliations:** 1 Department of Systems Biology, College of Bioinformatics Science and Technology, Harbin Medical University, Harbin, China; 2 Department of Radiology, Harbin Medical University Cancer Hospital, Harbin, China; 3 The Sino-Russian Medical Research Center of Jinan University, The Institute of Chronic Disease of Jinan University, The First Affiliated Hospital of Jinan University, Guangzhou, China

**Keywords:** Chromatin regulators, Synthetic lethality, Synthetic viability, Cancer therapy, Drug response

## Abstract

**Background:**

Diverse drug vulnerabilities owing to the Chromatin regulators (CRs) genetic interaction across various cancers, but the identification of CRs genetic interaction remains challenging.

**Methods:**

In order to provide a global view of the CRs genetic interaction in cancer cells, we developed a method to identify potential drug response-related CRs genetic interactions for specific cancer types by integrating the screen of CRISPR-Cas9 and pharmacogenomic response datasets.

**Results:**

Totally, 625 drug response-related CRs synthetic lethality (CSL) interactions and 288 CRs synthetic viability (CSV) interactions were detected. Systematically network analysis presented CRs genetic interactions have biological function relationship. Furthermore, we validated CRs genetic interactions induce multiple omics deregulation in The Cancer Genome Atlas. We revealed the colon adenocarcinoma patients (COAD) with mutations of a CRs set (*EP300*, *MSH6*, *NSD2* and *TRRAP*) mediate a better survival with low expression of *MAP2* and could benefit from taxnes. While the COAD patients carrying at least one of the CSV interactions in Vorinostat CSV module confer a poor prognosis and may be resistant to Vorinostat treatment.

**Conclusions:**

The CRs genetic interaction map provides a rich resource to investigate cancer-associated CRs genetic interaction and proposes a powerful strategy of biomarker discovery to guide the rational use of agents in cancer therapy.

**Supplementary Information:**

The online version contains supplementary material available at 10.1186/s12967-022-03651-w.

## Introduction

Chromatin regulators (CRs) are indispensable upstream regulatory factors in establishing and maintaining epigenomic landscape [1]. According to their specialized functions in epigenetics, CRs can be categorized into three major subgroups: DNA methylators, histone modifers, and chromatin remodelers [2]. Genomic alteration of CRs is a prevalent feature in various cancer types [1]. Cancers with deficiency in CRs promote cancer cells to rewire transcriptional regulatory circuits, affect the expression of downstream regulatory factors. A growing number of dysfunction CRs have been recognized as novel therapeutic targets in cancers and offered new therapeutic opportunities [3, 4]. For example, *ARID1A*, a component of SWI/SNF chromatin-remodeling factor, is one of the most frequently mutated genes in various cancers. Furthermore, *ARID1A*-deficient cancer cells are specifically vulnerable to *EZH2* inhibitors, *HDAC* inhibitors and some other selective inhibitors [5].

Genetic interaction is generated when pairwise perturbations of two genes result in an unexpected phenotypic outcome. According to the diverse impact in phenotype, genetic interaction can be further divided into synthetic lethality and synthetic viability. A synthetic lethal interaction describes the scenario in which the deficiency of single gene is viable, while a combination of alterations in two genes simultaneously induces cell death, which can be applicable to identify drug targets and pharmaceutical sensitive biomarkers [6]. Synthetic lethality has emerged as an attractive therapeutic strategy, especially the success of poly (ADP-ribose) polymerase 1 (*PARP1*)-targeted therapy against the growth of *BRCA1/2*-mutated cancer cells [7]. Synthetic viability interaction refers to dysfunction of both genes that can rescue the lethal effects of the individual gene alterations. Our previous work revealed that synthetic viability could induce drug resistance in cancer cells [8]. Therefore, the identification of cancer viability-related genomic biomarkers should be conductive to marked improvements in drug response and cancer therapy.

Genetic interaction analysis in cancer genome revealed that dysfunction CRs may have potential synthetic effect with other genes and play major roles in cancer therapy [8]. However, rare studies focused on systematically detecting CRs synthetic lethality (CSL) interaction and CRs synthetic viability (CSV) interaction in cancer genome, especially their roles on drug response of cancer cells. Functional perturbation technologies, such as short hairpin RNA (shRNA) and CRISPR-Cas9 have offered effective tools to screen genetic interaction for human cancer cells [9]. An integration of pharmacogenomic datasets provided a robust and reliable implement to predict the CRs genetic interaction related to pharmacological response in cancer cells [10].

**Fig. 1:**
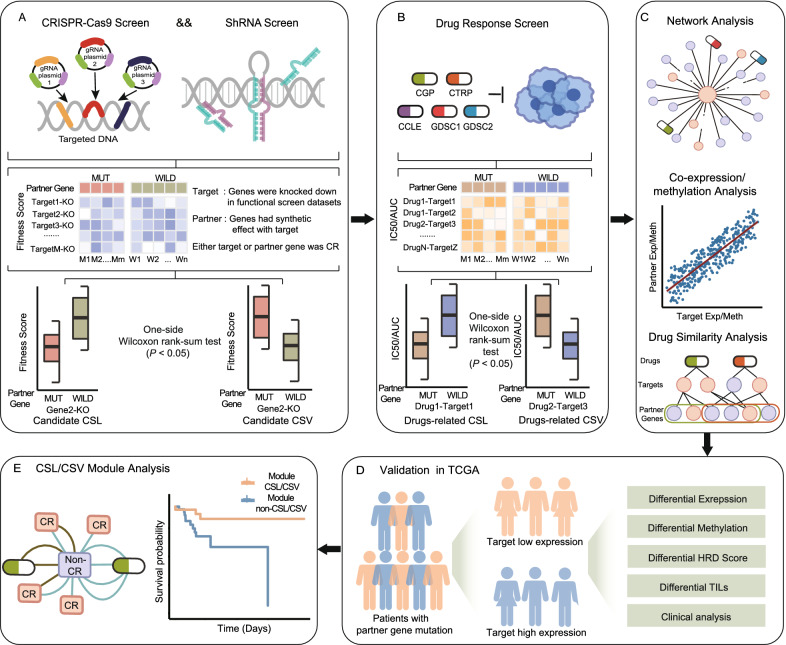
Workflow of this work. **A** Identification of CSL and CSV. **B** Screening drug response-related CRs genetic interaction based on pharmacogenomic datasets. **C** Analysis of CRs genetic interaction network. **D** Evaluation of CSL or CSV interaction in TCGA datasets. **E** CSL or CSV module analysis in COAD

In the present study, we developed an algorithm to identify CRs genetic interaction according to the coalition of functional screen data and investigate the drug response effect using pharmacogenomic datasets (Fig. 1A and Fig. 1B). The CRs genetic interaction network analysis indicated CSL and CSV interaction may be conducive to candidate biomarkers for cancer therapies (Fig. 1C). By mining drug response-related CR genetic interactions in The Cancer Genome Atlas (TCGA), we inspected their special effect in transcriptional control, epigenetic changes, genomic instability, tumor microenvironment and survival outcome (Fig. 1D and Fig. 1E). Moreover, the biomarkers identified by our work will conductive to predict the mechanism of drug response in cancer treatment and will guide precise targeting of clinical application.

## Methods

### Identification of candidate CRs genetic interactions

CRs with documented functions were collected by manual curation [2, 3]. A comprehensive list of drug targets was compiled from DrugBank (https://go.drugbank.com/) and some other public pharmacogenomic datasets.

In the present work, we collected and exploited three functional screen data, two high-throughput CRISPR-Cas9 screening datasets and one shRNA data, to identify candidate genetic interaction related to CRs (Additional file 1: Table S1). CRISPR1 screening data, including 625 cell lines, were obtained from DepMap Portal (https://depmap.org/portal/) (version 19Q3). DepMap Portal used the Avana CRISPR-Cas9 genome-scale knockout library and CERES algorithm to identify genetic vulnerabilities of cancer cells. Genes with lower scores represent that they are more essential [11]. Parental cell lines and mutation data were downloaded from the Cancer Cell Line Encyclopedia project (CCLE, https://portals.broadinstitute.org/ccle) [12].

Another CRISPR-Cas9 fitness screen with 324 cell lines, CRISPR2, was downloaded from DepMap ProjectScore (https://score.depmap.sanger.ac.uk/). Genome-scale CRISPR-Cas9 fitness scores indicate the ability required for cell viability [13]. Higher fitness scores indicated proficiency of cell growth and viability. Mutation data was obtained from Catalogue of Somatic Mutations in Cancer (COSMIC, https://cancer.sanger.ac.uk/cosmic).

We downloaded shRNA data from DepMap Portal, Achilles 2.20.2, which provides gene knockdown viability effects (gene dependency scores) across 501 cancer cell lines. Higher shRNA scores indicate enrichment of cell viability [14]. The genetic background of cell lines was downloaded from CCLE [12].

Only tissue types with more than three cell lines were included for further analyses in individual dataset. For each pair of genes (target and partner, either target or partner gene was CRs), fitness scores of target knockdown were compared between the cell lines with and without partner alterations (or multi-mutation types) using one-sided Wilcoxon rank-sum test. Provided that the fitness scores of partner mutation cells are lower/higher than wild type samples (*P* < 0.05) when target is knocked down, partner and target were predicted as candidate CSL or CSV genetic interaction.

For each candidate genetic interaction observed in at least two procedures, we combined the *P* values obtained by each inference procedure into a single *P* value via Fisher's combined probability test in metaseqR R package [15]. The combined *P* value with *P* < 0.05 was used to further analysis.

Quantitative profiling of proteins by mass spectrometry across 375 cell lines from diverse lineages was downloaded from DepMap Portal [16]. The correlation between several protein expressions of mutated genes and the target genes dependency were computed in lung cancer cell lines via Pearson's correlations analysis.

### Screening CSL and CSV interaction related to drug response

Five drug datasets were applied to filter drug biomarkers in cancer cells (Additional file 1: Table S2). The original pharmacological screening data were downloaded from CCLE [12] (https://portals.broadinstitute.org/ccle), Cancer Therapeutics Response Portal [17, 18] (CTRP, https://www.broadinstitute.org/ctrp/), Cancer Genome Project [19] (CGP) and another two datasets from Genomics of Drug Sensitivity in Cancer[20] (GDSC, https://www.cancerrxgene.org/). CCLE dataset is constituted of the half maximal inhibitory concentration (IC50) values of 24 anti-cancer compounds used to treat 504 cancer cell lines. CGP dataset contained the IC50 of 130 drugs across 639 cancer cell lines; exceptionally low IC50 values from a cell-based assay were achieved along with remarkably high therapeutic indices. The CTRP data contained the area-under-concentration-response curve (AUC) of 481 small molecules across 835 cancer cell lines. As a measure of cancer cell lines sensitivity to small molecule treatment, a low AUC usually indicates high sensitive in the cell lines. The GDSC1 data contained AUC values of 304 drugs detected in 988 cell lines. The GDSC2 data contained AUC values of 169 drugs detected in 811 cell lines. The cell line information and mutation data of GDSC and CGP datasets were referenced from COSMIC, and information related to CTRP and CCLE was available from CCLE. Only tissue types with more than three cell lines treated by the same compounds were included for further analyses in individual dataset.

If target in the CSL or CSV pair was targeted or affected by a drug, the IC50/AUC of cell lines with and without alterations in partner gene were compared using a one-sided Wilcoxon rank-sum test (*P* < 0.05). If the IC50/AUC of partner mutated cells are lower or higher than wild type samples (*P* < 0.05), the genetic interactions between the target and partner gene were identified as drug response-related CSL or CSV genetic interaction.

### Generation of CRs interaction network and functional analysis

The network of drug response-related CSL or CSV were constructed and visualized by Cytoscape software (https://cytoscape.org/). Protein-protein interaction (PPI) data was obtained from the Pathway Commons database [21] (http://www.pathwaycommons.org/).

We randomly selected the same number of gene pairs from the genes having interactions in Pathway Commons and calculated the rate of direct or indirect interaction that overlapped with the PPI networks. The empirical *P* value was counted according to 1000 random CSL and CSV networks.

Gene expression, DNA methylation cohorts of TCGA were derived from the UCSC Xena database (http://xena.ucsc.edu/). Pearson's correlations were used for the statistical analysis of co-expression and co-methylation. The statistical *P* value was less than 0.05.

Cancer related genes were downloaded from the cancer gene census [22] (http://cancer.sanger.ac.uk/census).

### The similarity analysis of drug pairs

We hypothesized that similar drugs share common partner genes and divided the drug pairs into similar groups and non-similar groups, for paired drugs in CSL interaction and in CSV interaction by hypergeometric test (*P* < 0.05).

Clinical similarities of drug pairs derived from the drug Anatomical Therapeutic Chemical (ATC) classification systems codes were used to predict new drug targets [23]. The ATC codes for drugs used in this study were obtained from NCBO BioPortal [24]. Jaccard similarity coefficient was calculated to evaluate the similarity of paired drugs.

We also computed Gene Ontology (GO) similarity for each pair of targets in paired drugs by a graph-based semantic similarity measure algorithm implemented in GOSemSim R package [25]. GO similarity score contained three type of score, biological processes (BP), molecular function (MF), and cellular component (CC).

### Functional analysis of the genes interacting with CRs in CSL and CSV interaction

A hypergeometric distribution model was used to test whether the the genes interacting with CRs in CSL and CSV network were significantly enriched in biological pathways from the Kyoto Encyclopedia of Genes and Genomes (KEGG) database [26]. The statistical *P* value was corrected by the Benjamini and Hochberg (BH) correction for multiple tests, with cutoffs of *False discovery rate* (*FDR*) < 0.1 established for significant pathways.

### Differential expression/methylation/peak accessibility/HRD/TIL analysis

Gene expression, DNA methylation, mutation, Assay for transposase-accessible chromatin with sequencing (ATAC-seq) peak signal [27] and homologous recombination deficiency (HRD) score cohorts of TCGA were derived from the UCSC Xena database (http://xena.ucsc.edu/). The overall distribution of immune cell fractions in TCGA cancers were systemically inferred by CIBERSORT [28] (https://cibersort.stanford.edu/).The patients with partner gene mutation were divided into two groups, according to the median expression level of the targets. The partner genes with mutation frequency more than 10% were included in the analysis. One-sided Wilcoxon rank-sum test were used to identify the differential expression mRNA and methylation gene, as well as differential accessibility of peaks. The statistical *P* value was corrected by the BH correction for multiple tests, and the *FDR* < 0.1. One-sided Wilcoxon rank-sum test were used to identify the differential HRD score or immune microenvironment, with cutoffs of *P* < 0.05.

### Survival analysis

The mutation and mRNA expression profiles of TCGA were analyzed to examine the prognostic value embedded in the CSL and CSV networks. For individual CSL or CSV, the patients with partner gene mutation were divided into two groups according to the median expression value of the targets in CSL or CSV. For the accumulated effect of CSL or CSV interaction, the patients were divided into three groups according as the number of individual CSL or CSV interactions they carried. The log-rank test was used to assess the survival difference in specific cancer types. We presented the results by Kaplan Meier plots.

## Results

### Generation of a drug response-related CRs genetic interaction map in cancer cells

**Fig. 2:**
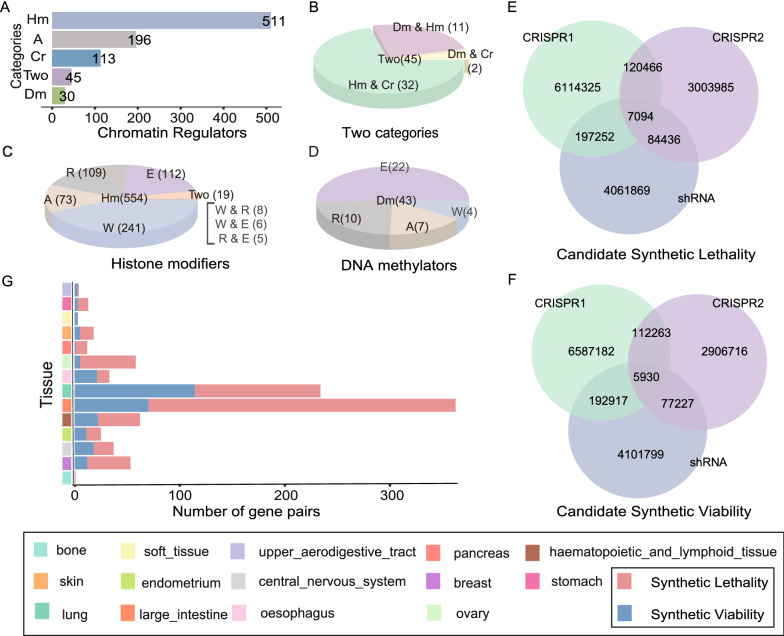
Identification of CSL and CSV interactions in cancer cells. **A** Bar charts show the numbers of CRs in different categories. **B** Specific roles of the CRs with two functions, including Dm & Hm (DNA methylation and histone modification), Dm & Cr (DNA methylation and chromatin remodeling), Hm & Cr (histone modification and chromatin remodeling). **C** Subgroups of the histone modifiers. **D** Subgroups of the DNA methylator. Dm, Hm and Cr depicts DNA methylator, histone modifier and chromatin remodeler. Two means two types, including W & R (Writer and Reader), W & E (Writer and Eraser) and R & E (Reader and Eraser). A denotes ambiguous. R, W and E represent reader, writer and eraser. **E** Overlapping of the candidate CSL interactions identified in functional screen datasets. **F** Overlapping of the candidate CSV interactions identified in functional screen datasets. **G** Statistics of CSL and CSV in different types of cancer cells

A set of 895 CRs with documented functions in the following chromatin regulation procedures: histone modification, chromatin remodeling, DNA methylation and some genes of unknown function were complied (Fig. 2A). There are 45 genes playing multiple roles in chromatin regulation (Fig. 2B). Histone modifiers and DNA methylators can be further divided into three subgroups: reader, writer and editor (Fig. 2C and Fig. 2D). 18490 drug and targets relationships were are composed of 5289 drugs and 2454 targets. We identified candidate CSL or CSV based on a combination of CRISPR-Cas9 and Achilles shRNA dataset (Fig. 1A; Additional file 1: Table S1). The strategy was based on the notion that knockdown of target causes a selective reduction (CSL) or enhancement (CSV) in cell viability, with simultaneous mutation in another partner gene. Only these gene pairs with a united effect detected by at least two of the three functional screen datasets and with combined *P* value less than 0.05 were selected for further analysis. According to the screening criteria, 409248 candidate CSL interaction (Fig. 2E) and 388337 candidate CSV interaction (Fig. 2F) were identified in tissue-specific cancer cell lines.

Five drug datasets were used to predict drug biomarkers by investigating the CSL and CSV interaction in cancer cells with specific drugs treatment (Fig. 1B; Additional file 1: Table S2). For CSL interaction between drug target and partner gene, we tested whether cell lines with mutations of the partner gene were more sensitive than the cell lines with wild-type partner gene to the drug (*P* < 0.05, one-sided Wilcoxon rank-sum test). For CSV interaction between drug target and partner gene, we tested whether cell lines with mutations of the partner gene were more resistant than the cell lines with wild-type partner gene to the drug (*P* < 0.05, one-sided Wilcoxon rank-sum test). Ultimately, we revealed 625 CSL interactions (Additional file 2: Table S3), as well as 288 CSV interactions (Additional file 2: Table S3) in tissue-specific cancer cells, which have potentiality to predict drug response (Fig. 2G). For example, we found *RB1* mutation was associated with resistance to Palbociclib, a highly selective inhibitor of *CDK4/6*, in lung cancer cells (Additional file 1: Fig. S1). Thangavel et al*.* found that *CDK4/6* inhibition results in apoptosis specifically in *RB*-proficient non-small cell lung cancer (NSCLC) models, but did not impact growth on *RB*-deficient tumors [29].

### Drug response-related CRs genetic interaction network shows biological characteristics

**Fig. 3:**
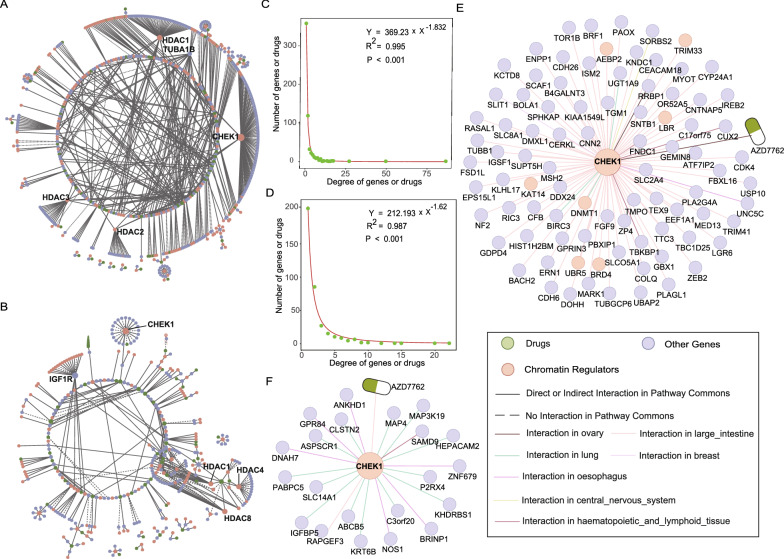
Analysis of CRs genetic interaction networks. **A** Drug response-related CSL interaction network. **B** Drug response-related CSV interaction network. **C** Distribution of the degree of genes or drugs in the CSL network. **D** Distribution of the degree of genes or drugs in the CSV network. **E** *CHEK1* subnetwork in drug response-related CSL network. **F** *CHEK1* subnetwork in drug response-related CSV network

Drug response-related CSL and CSV networks were constructed, respectively. The CSL interaction network was constituted of 552 genes, 202 CRs included, as well as 67 drugs (Fig. 3A). 328 genes, 134 CRs included, and 54 drugs comprised the CSV interaction network (Fig. 3B). The percentage of cancer genes in CRs genes increased in CRs genetic interaction networks than the primary 895 CRs, either in CSL (*P* = 0.05, Chi-square test) or CSV networks (*P* = 8.9E−3, Chi-square test), which suggest CRs genetic interaction is closely related to cancers. 83% of CSL interactions and 75% of CSV interactions have direct or indirect contact in Pathway Commons, which cannot expected by random chance *(P* < 1E−3; Fig. 3A and B). Both CSL and CSV networks showed scale-free characteristics (Fig. 3C and D). The aforementioned results indicate that the CSL and CSV networks have potential biological functions.

In CSL and CSV networks, the top 5 genes with highest degree were marked with symbols (Fig. 3A and B). Some CR genes, such as *CHEK1* and histone deacetylase (*HDAC*) family, had high degrees in both CSL and CSV network. Nevertheless, the hubs interacted with distinct partner genes across various cancer cells or were influenced by different drugs (Fig. 3E and F). *CHEK1* was inhibited by AZD7762, and the mutation of some sensitive genes, such as *BRF1*, *CDK4* and *MSH2*, associated with a worse viability to large intestine cancer cells (Additional file 1: Fig. S2). Several studies have reported that mismatch repair genes *MSH2* deficiency could enhance tumor proliferation via the *ATR-CHEK1* pathway in pituitary adenoma [30]. Interestingly, lung cancer cells with mutations of resistant genes, such as *ABCB5*, *IGFBP5*, *MAP4*, showed high proliferation treatment by drug AZD7762 (Additional file 1: Fig. S3). We highlighted the variation in synthetic lethal/viable effects observed in different cancer cells in consideration of the major genetic heterogeneity.

### CRs genetic interactions have biological function relationship

**Fig. 4:**
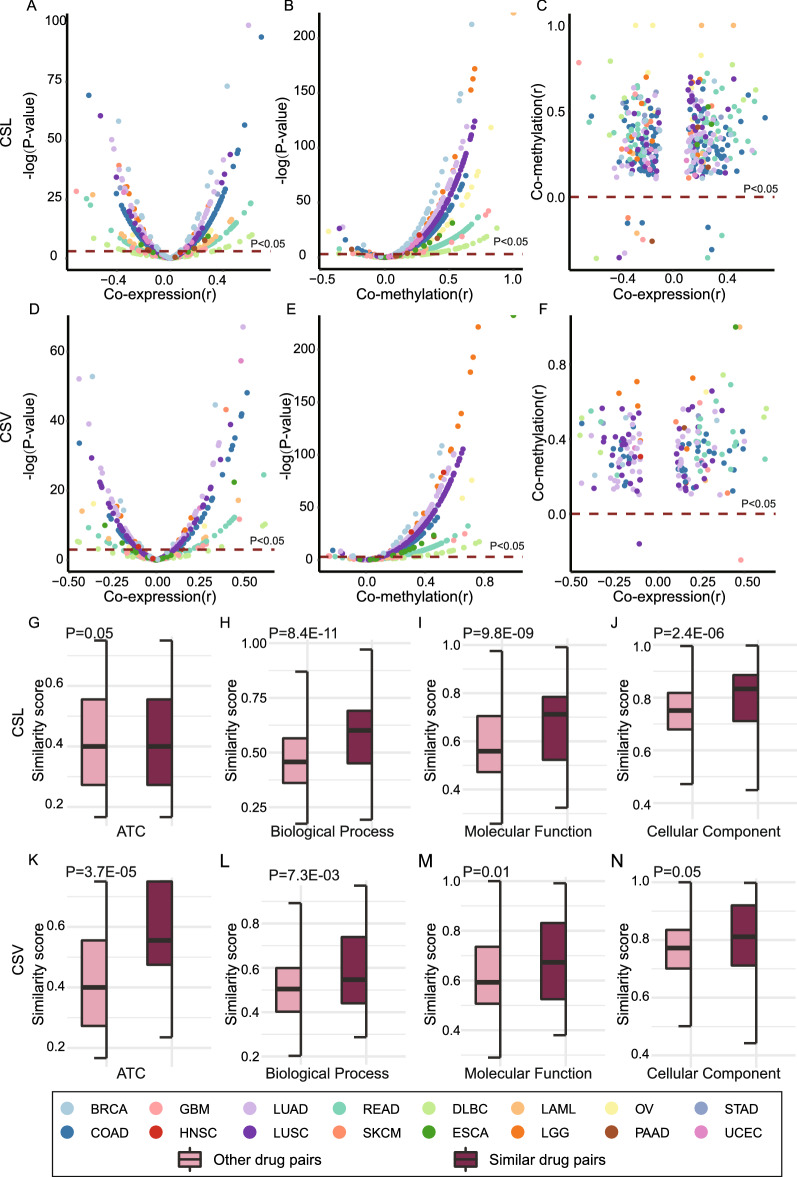
Functional analysis of CRs genetic interactions. The co-expression analysis for CSL interaction (**A**) and CSV interaction (**D**) were investigated in TCGA. The co-methylation analysis for CSL interaction (**B**) and CSV interaction (**E**) were investigated in TCGA. The co-expressed interaction and co-methylaetd interaction were detected, respectively for CSL (**C**) and CSV (**F**). The drug therapeutic similarity score of similar drug pairs and other drug pairs in CSL (**G**) and CSV (**K**) map were showed. Similarity of drug targets in terms of BP (**H**), MF (**I**) and CC (**J**) in CSL map was detected. Similarity of drug targets in terms of their BP (**L**), MF (**M**) and CC (**N**) in CSV map was detected. *P* values were calculated by t test in G-N

In addition, we discovered gene pairs in CSL or CSV tended to be co-expressed or co-methylated in the tissue-specific cancer types from TCGA. More than 25% of CSL interaction showed positive expression correlation; 21% of CSL interaction were negatively correlated at mRNA expression level (Fig. 4A). 75% of the CSL showed positive correlation at methylation level, however, only 2% CSL gene pairs showed negative correlation at methylation level (Fig. 4B). There was a positive correlation for 27% of the CSV interaction at mRNA expression level as well as 21% were negative correlation (Fig. 4D). Also, 77% of the CSV interactions displayed positive correlation and less than 2% was negative at the methylation level (Fig. 4E). Notably, the genetic interactions with co-expression tended to exhibit more positive co-methylation, either in CSL or in CSV interactions (Fig. 4C and F). Thus, these results suggest that genetic interactions present biological regulation relationship not only in cell lines, but also in tissue-specific cancers.

Kyoto Encyclopedia of Genes and Genomes (KEGG) pathway enrichment analysis was performed to investigate the biological pathways of non-CR genes involved in CSL and CSV interaction network. Genes interacting with CRs were enriched with 57 and 50 non human diseases pathways of KEGG, respectively in CSL and CSV interactions (*FDR* < 0.1, hypergeometric test; Additional file 1: Fig. S4A and B). The overlapping pathways, such as cell cycle, *PI3K-AKT* signaling pathway and *Rap1* signaling pathway, are cancer hallmark signaling pathways (Additional file 1: Fig. S4C). The aforementioned results indicate that biomarkers have synthetic lethal and synthetic viable effect with CRs by disturbing cancer related signaling pathways.

There were 67 drugs and 54 drugs involved in the CSL and CSV interaction. Under the hypothesis that similar drugs often share similar partner genes, the similar drug pairs were defined when two drugs have significantly overlap between their partner genes. We identified 137 similar drug pairs and 2074 other drug pairs in CSL interaction network. For example, vinblastine and vincristine, both of which are vinca alkaloids drugs, shared 8 partner genes in CSL interaction network (*P* < 1.0E−6, hypergeometric test). Also, the similar drugs defined by our work had higher drug therapeutic similarity score based on the Anatomical Therapeutic Chemical Classification System (ATC) code (Fig. 4G). According to the GO annotations, the targets of similar drug pairs had higher similarity score in terms of their BP (Fig. 4H), MF (Fig. 4I) and CC (Fig. 4J). Moreover, 52 similar drug pairs and 1379 other drug pairs were identified in CSV interaction network. Two epidermal growth factor receptor (EGFR)-targeted agents afatinib and erlotinib shared significantly partner genes in the CSV interaction network (*P* = 9.4E−4, hypergeometric test). The similar drug groups also exhibited higher ATC similarity score (Fig. 4K) and GO similarity score (Fig. 4L, M and N). Thus, our drug response-related CRs genetic interaction map proposes a reliable measure of drug-drug relationships and may contribute to drug reposition.

### CRs genetic interactions induce multiple omics deregulation in TCGA

**Fig. 5:**
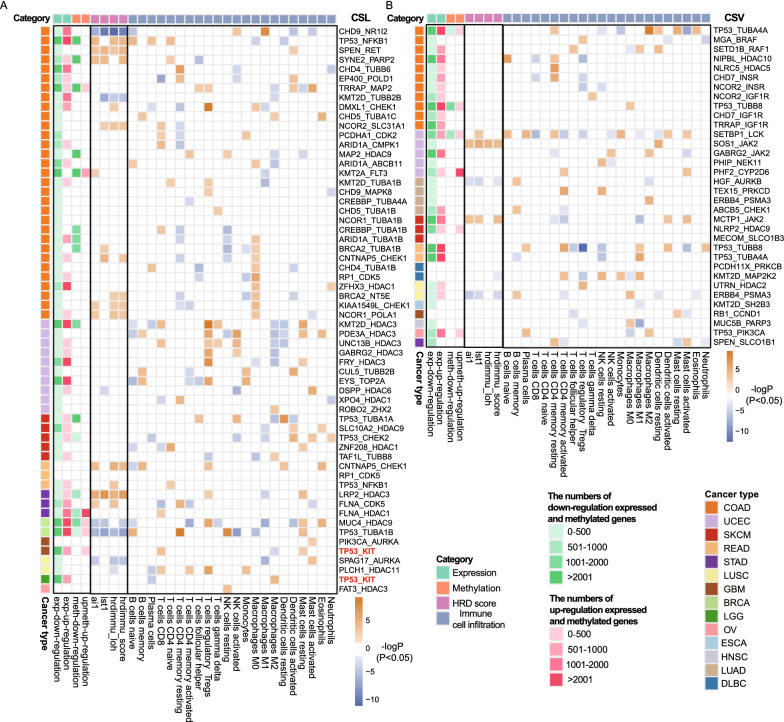
CRs genetic interactions induce multiple omics deregulation in TCGA. **A** CSL interactions disrupt gene expression, DNA methylation, homologous recombination repair ability and immune cell infiltration in TCGA. **B** CSV interactions disrupt gene expression, DNA methylation, homologous recombination repair ability and immune cell infiltration in TCGA. Red or green entries represent the number of up-regulated or down-regulated genes and hyper-methylated or hypo-methylated DNA. Differentially methylated genes or differentially expressed gene were identified using one-sided Wilcoxon rank-sum test with *FDR* < 0.1. Yellow entries indicate up-regulation of the HRD score or TIL, and purple entries imply down regulation of the HRD score or TIL. Heatmap of the HRD score and TIL shows the –log*P*, where *P* values were calculated by one-sided Wilcoxon rank-sum test

CRs regulate localized or globalized epigenome and transcriptome to affect multiple target genes [3]. For a CRs genetic interaction pair, the cancer patients with mutations of the partner genes were divided into two groups according to the expression of the drug target, where low expression of the drug target mimicked the drug inhibition effect. And, we tested the difference of mRNA expression, DNA methylation, homologous recombination repair ability and immune cell infiltration between the two groups. On the precondition of partner genes mutation, targets expression level significantly disrupted a vast number of genes mRNA expression and DNA methylation in CSL (*FDR* < 0.1, one-sided Wilcoxon rank-sum test; Fig. 5A) and CSV (*FDR* < 0.1, one-sided Wilcoxon rank-sum test; Fig. 5B) interactions. More than 91% (55/61) of CSL interactions deregulated gene expression and more than 26% (16/61) of CSL interactions disrupted DNA methylation. 88% (30/34) of CSV interactions deregulated gene expression and more than 17% (6/34) of CSV interactions disrupted DNA methylation. For example, *TP53* mutation has synthetic lethal effect with the inhibition of *KIT* by Dasatinib. In glioblastoma (GBM) or low-grade glioma (LGG) with *TP53* mutations, 3137 and 3648 genes were significantly differentially expressed in the *KIT* down-regulation GBM or LGG group compared with *KIT* up-regulation GBM or LGG group (Additional file 1: Fig. S4D). The overlapping between the two sets of differentially expressed genes could not be expected by random chance (*P* < 1E−16, hypergeometric test). The overlapping differentially expressed genes were significantly enriched in ErbB signaling pathway (*P* = 1.8E−04, hypergeometric test), *Ras* signaling pathway (*P* = 1.5E−05, hypergeometric test) and *Rap1* signaling pathway (*P* = 9.8E−04, hypergeometric test; Additional file 1: Fig. S4E). Above results suggest that mutations of *TP53* may have synthetic lethal effect with the inhibition of *KIT* by disrupting cancer related signaling pathways.

To better understand epigenomic landscape differences between patients with and without CSL/CSV interactions, we analyzed differential chromatin accessibility for breast cancer (BRCA) and colon adenocarcinoma (COAD) (Additional file 1: Fig. S5A). We revealed distinct patterns of genome-wide chromatin accessibility between patients with and without CSL/CSV interactions. We found 1423 differentially accessible peaks between BRCA patients with and without *TP53-TUBA1B* CSL interaction and 4120 differentially accessible peaks for BRCA patients with and without *MUC4-HDAC9* CSL interactions (Additional file 1: Fig. S5B and C). In COAD, 4331 peaks showed increased accessibility and 4040 showed decreased accessibility peaks in patients with *TP53-NFKB1* CSL interaction (Additional file 1: Fig. S5D). In addition, there were 278 and 500 differentially accessible peaks for *TP53-TUBA4A* and *TP53-TUBB8* of which had CSV interactions in COAD, respectively (Additional file 1: Fig. S6A and B). Above results reveal CRs genetic interactions could induce distinct chromatin accessibility.

More than 36% (22/61) of CSL interactions deregulated homologous recombination repair ability and more than 95% (58/61) of CSL interactions disrupted immune cell infiltration (Fig. 5A). 17% (6/34) of CSV interactions deregulated homologous recombination repair ability and more than 85% (29/34) of CSV interactions disrupted immune cell infiltration (Fig. 5B). For example, HRD scores were upregulated under the influence of eight CSL interactions in COAD. In uterine corpus endometrial carcinoma (UCEC), three CSL interactions significantly promoted memory B cells infiltrating and five CSL interactions downregulated M2 Macrophages. Memory B cells and M2 Macrophages contribute to a collective function in synthetic lethality. Above results indicate that CSL or CSV genetic interactions have effects on drug response by interfering DNA repair ability and tumor microenvironment in cancer.

### CRs genetic interaction mediate distinct drug response and survival outcomes

**Fig. 6:**
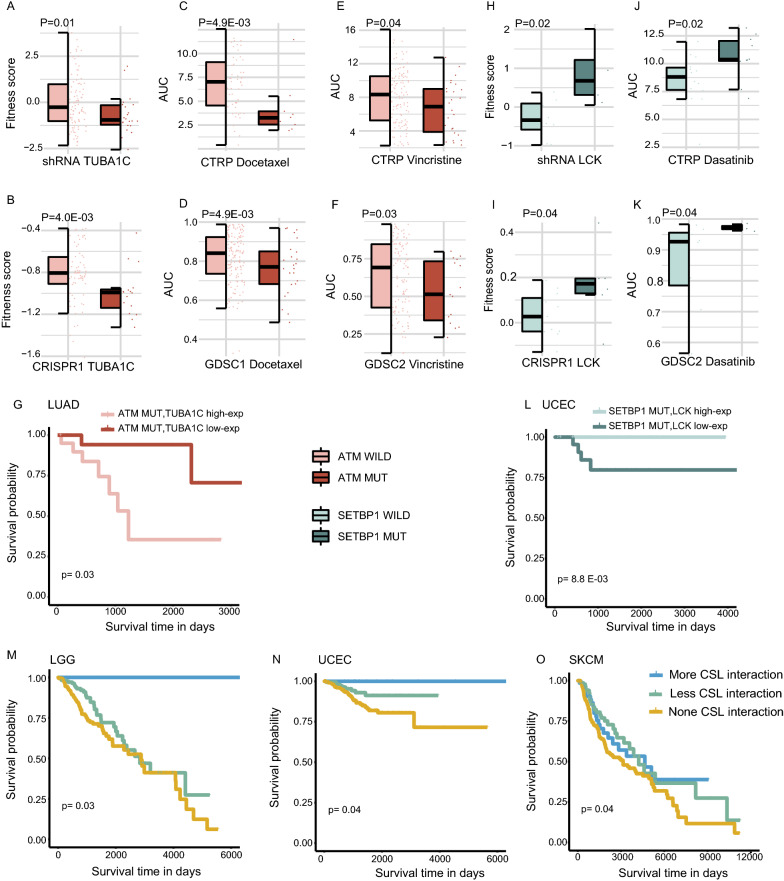
CRs genetic interaction mediate distinct drug response and prognosis. Lung cancer cell lines with *ATM* mutation have worse viability when *TUBA1C* were knocked down in shRNA (**A**) and CRISPR1 (**B**). Cell lines with *ATM* mutation are sensitive to Docetaxel in CTRP (**C**) and GDSC1 (**D**). Cell lines with *ATM* mutation are sensitive to Vincristine in CTRP (**E**) and GDSC2 (**F**). The Kaplan–Meier overall survival of *ATM* mutation carriers in LUAD patients in two groups as follows: *TUBA1C* low-expression and *TUBA1C* high-expression (**G**). Endometrium cell lines with *SETBP1* mutation have better viability when *LCK* were knocked down in shRNA (**H**) and CRISPR1 **(I)**. Endometrium tissues with *SETBP1* mutation are resistant to Dasatinib in CTRP (**J**) and GDSC2 (**K**). The Kaplan–Meier overall survival of *SETBP1* mutation carriers in UCEC patients in two groups as follows: *LCK* low-expression and *LCK* high-expression (**L**). The cumulative effect of CSL interaction induces a better prognosis in LGG (**M**), UCEC (**N**) and SKCM (**O**). *P* values in **A**–**F** and **H**–**K** were calculated by one-sided Wilcoxon rank-sum test. *P* values in G, L, M, N and O were tested by log-rank test

*TUBA1C*, an oncogene and key microtubule component implicated in multiple cancers, was targeted by Docetaxel and Vincristine [31]. *TUBA1C* interacted with 5 CRs in drugs related CSL interaction network across 3 tissues (Additional file 1: Fig. S7). Pathway enrichment analysis indicated that the partner genes of *TUBA1C* in CSL interaction network were mainly enriched in the *p53* signaling pathway, Lysine degradation and apoptosis pathway (*P* < 0.05, hypergeometric test; Additional file 1: Fig. S7). For example, knock-down *TUBA1C* by shRNA in lung cancer cells with *ATM* mutation showed a worse survival than cells with wild-type *ATM* (*P* = 0.01, one-sided Wilcoxon rank-sum test; Fig. 6A). A similar results can be identified in CRISPR1 dataset (*P* = 4.9E−03, one-sided Wilcoxon rank-sum test; Fig. 6B). Furthermore, *ATM* mutation cell lines were linked with Docetaxel sensitivity and Vincristine sensitivity in lung tissues (*P* < 0.05, one-sided Wilcoxon rank-sum test; Fig. 6C, D, E and F). *ATM* frequently alters in various cancers and plays a crucial role in numerous DDR-regulated cellular responses, such as DNA repair, apoptosis and cell cycle arrest [32]. The *ATM* mutated patients with *TUBA1C* low expression showed better survival than patients with over-expression of *TUBA1C* in lung adenocarcinoma (LUAD) (*P* = 0.03, log-rank test; Fig. 6G). These results suggest that LUAD patients with *ATM* mutation may be beneficial from Docetaxel and Vincristine. In addition, other CSL interactions also have the potentiality to be a survival prediction biomarker, such as *TP53* and *KIT* in LGG (*P* = 0.01, log-rank test; Additional file 1: Fig. S8A) as well as *BCORL1* and *GSTP1* in LUAD (*P* = 0.01, log-rank test; Additional file 1: Fig. S8B).

In contrast to CSL interaction, CSV interaction mediated drug resistance and a worse prognosis. For example, *LCK* had synthetic viability effect with *SETBP1*. A enhancement of cell growth or viability were identified when *LCK* was knocked down by shRNA (*P* = 0.02, one-sided Wilcoxon rank-sum test; Fig. 6H) or CRISPR1 (*P* = 0.04, one-sided Wilcoxon rank-sum test; Fig. 6I) in endometrium tissue with mutations of *SETBP1*. In addition, *SETBP1* mutated endometrium cancer cells were resistant to Dasatinib, a targeted drug of *LCK* in CTRP (*P* = 0.02, one-sided Wilcoxon rank-sum test; Fig. 6J) and GDCS2 (*P* = 0.04, one-sided Wilcoxon rank-sum test; Fig. 6K). In UCEC patients with mutations of *SETBP1*, individuals with low-expression of *LCK* showed worse prognosis than those with high-expression of *LCK* (*P* = 8.8E−03, log-rank test; Fig. 6L). Besides that, other CSV interactions also can be a predictive biomarker in other cancers, such as *BRWD3* and *PSMB8* in LUAD (*P* = 8E−04, log-rank test; Additional file 1: Fig. S8C), as well as *CNTN2* and *HDAC11* (*P* = 0.01, log-rank test; Additional file 1: Fig. S8D) in LUAD. The aforementioned results indicate the possibility and reliability of our CSV interaction in survival prediction of tumor patients.

### The accumulation of CSL interaction confer favorable prognosis

As we known, the accumulation of genetic interactions conferred to the prognosis of cancer patients [33]. The cancer patients were divided into three groups according to the number of activated CSL pairs within each patient: none, fewer interactions (less than the median number of interactions in patients) and more interactions (greater than the median number of interactions in patients). As expected, cancer patients with more activated CSL pairs showed better prognosis in LGG (*P* = 0.03, log-rank test; Fig. 6M), UCEC (*P* = 0.04, log-rank test; Fig. 6N) and SKCM (*P* = 0.04, log-rank test; Fig. 6O). For CSV interaction, the opposite result can be detected in esophageal cancer (ESCA). The ESCA patients with at least one of the activated CSV gene pairs had poorer prognosis than patients without activated CSV pairs (*P* = 0.13, log-rank test; Additional file 1: Fig. S8E).

### MAP2 CSL module mediate better prognosis in COAD

**Fig. 7:**
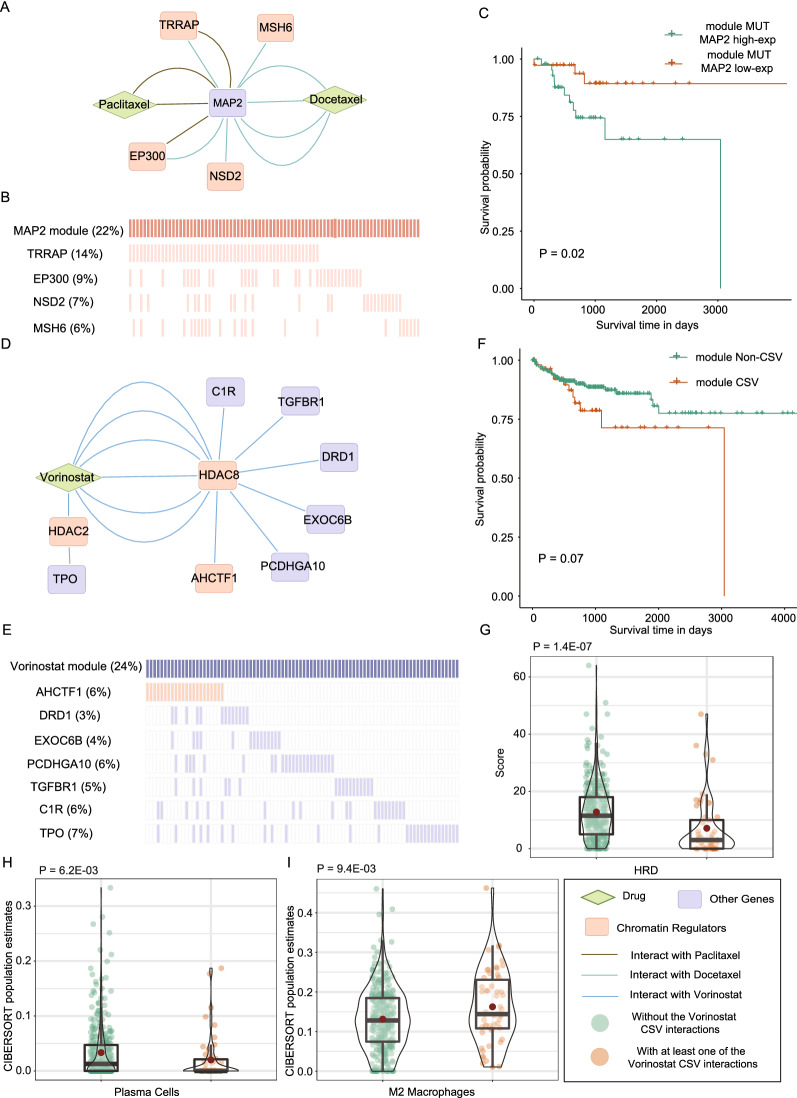
CSL and CSV module are related to distinct prognosis in COAD. **A** The CSL interactions with *MAP2* in COAD. **B** Mutation frequency of partner genes of *MAP2* in COAD. **C** The Kaplan–Meier survival analysis of classifications generated by *MAP2* expression in the patients with module partner genes mutation. *P* values were tested by log-rank test. **D** The CSV interactions with Vorinostat in large intestine cancer cells. **E** Partner genes mutation frequency in COAD. **F** The Kaplan–Meier survival analysis of classifications generated from the patients with and without Vorinostat CSV module interactions. **G** The comparison of HRD scores for the two subgroup in **F**. The distribution of Plasma cells (**H**) and M2 macrophages (**I**) infiltration for 2 subtypes in **F**. *P* values were calculated by one-sided Wilcoxon rank-sum test

Mutation frequency of individual partner genes in CSL or CSV interactions is quite low. To enhance the coverage of prediction range, we merged a module by integrating partner genes interacted with the same drug target in specific tissue. Four CRs (*TRRAP*, *EP300*, *NSD2* and *MSH6*) having synthetic lethal interactions with *MAP2* were integrated as a *MAP2* CSL module in COAD, where *MAP2* was affected by two taxnes (Paclitaxel and Docetaxel) (Fig. 7A).These four CRs play roles in cell cycle, notch signaling pathway and mismatch repair in human cancers. The mutation frequency of individual partners varied in 6%-14%, and the *MAP2* CSL module covers 22% samples in COAD (Fig. 7B). The accumulation of synthetic lethal effect in *MAP2* module mutations mediated a better prognosis in COAD patients with low expression of *MAP2* than COAD patients with high expression of *MAP2* (*P* = 0.02, log-rank test; Fig. 7C). However, no significant survival differences can be detected in the patients with wild-type of *MAP2* module genes (*P* = 0.80, log-rank test; Additional file 1: Fig. S9A). Next, using the fractions estimated by CIBERSORT, we found the patients with *MAP2* low expression or high expression showed significant difference in enrichment of tumor immune cell infiltration when *MAP2* module mutations. In COAD samples with mutations of *MAP2* module gene, the infiltration of M1 macrophages (*P* = 0.05, one-sided Wilcoxon rank-sum test; Additional file 1: Fig. S9B) and Resting Mast Cells (*P* = 0.02, one-sided Wilcoxon rank-sum test; Additional file 1: Fig. S9C) was lower in the COAD patients with *MAP2* low expression than those with *MAP2* high expression. The infiltration of Activated Mast Cells (*P* = 0.05, one-sided Wilcoxon rank-sum test; Additional file 1: Fig. S9D) and Regulatory T cells (Tregs; *P* = 0.03, one-sided Wilcoxon rank-sum test; Additional file 1: Fig. S9E) were higher in the COAD patients with *MAP2* low expression than those with *MAP2* high expression.

### Vorinostat CSV module confer poor prognosis in COAD

Vorinostat inhibits histone deacetylases (*HDAC*) family, including *HDAC2* and *HDAC8*. Inhibiting by Vorinostat, *HDAC8* had synthetic viable effects with six genes (*AHCTF1*, *DRD1*, *EXOC6B*, *PCDHGA10*, *TGFBR1* and *C1R*) and *HDAC2* had synthetic viable effects with *TPO* in large intestine tissue (Fig. 7D). Generally, the mutation frequency of individual partner genes was less than 8% (Fig. 7E). The partners mainly participate in *MAPK* signaling pathway, *TGF-beta* signaling pathway and *Hippo* signaling pathway. By integrating these partners as Vorinostat CSV module, the mutation frequency of module increased to 24%. The COAD individuals with at least one of the CSV interaction tended to have worse clinical outcome than the patients without these interactions in TCGA (*P* = 0.07, log-rank test; Fig. 7F). Differentially expressed genes between COAD patients with and without Vorinostat CSV interactions were significantly enriched in immune related biological processes, such as antigen processing presentation (*P* = 9.1E−08, hypergeometric test), B cell receptor signaling pathway (*P* = 1.6E−07, hypergeometric test) and T cell receptor signaling pathway (*P* = 1.1E−06, hypergeometric test).

In addition, the COAD individuals with at least one of the Vorinostat CSV module interactions showed lower genomic instability than the patients without Vorinostat CSV module interactions, including three independent DNA-based measures, telomeric allelic imbalance (TAI; Additional file 1: Fig. S10A; *P* = 8.0E−08, one-sided Wilcoxon rank-sum test), large-scale state transitions (LST; Additional file 1: Fig. S10B; *P* = 3.2E−05, one-sided Wilcoxon rank-sum test), loss of heterozygosity (HRD-LOH; Additional file 1: Fig. S10C; one-sided Wilcoxon rank-sum test, *P* = 1.2E−06) and the combined HRD score (Fig. 7G; *P* = 1.4E−07, one-sided Wilcoxon rank-sum test). High HRD score indicate genomic instability in the absence of homologous recombination. Therapies that target alternative DNA repair mechanisms, such as poly(ADP)-ribose polymerase inhibitors, have the potentiality to expand chemotherapy sensitivity and overcome drug resistance for COAD patients [34]. Besides genomic instability, the COAD patients carrying Vorinostat CSV interactions and the patients without Vorinostat CSV interactions showed distinct immune infiltration of tumor microenvironment. The infiltration of plasma cells was lower in the patients with Vorinostat CSV module interactions than the patients without Vorinostat CSV module interactions (Fig. 7H; *P* = 6.2E−03, one-sided Wilcoxon rank-sum test). The infiltration of M1 macrophages (Additional file 1: Fig. S10D; *P* = 5.4E−05, one-sided Wilcoxon rank-sum test), M2 macrophages (Fig. 7I; *P* = 9.4E−03, one-sided Wilcoxon rank-sum test) as well as Follicular helper T Cells (Additional file 1: Fig. S10E; *P* = 0.01, one-sided Wilcoxon rank-sum test) was distinctly higher in the patients with Vorinostat CSV module interactions than the patients without. M2 macrophages had been reported to pro-cancer roles in various types of cancers [35].

## Discussion

In the present work, we found mutations of CR genes have genetic interactions with mutations in cancer genome, which can affect the drug response in cancer cells. Totally, we identified 625 CSL interactions and 288 CSV interactions related to drug response. The majority of drug response-related CSL or CSV interactions have biological relationship revealed by protein interaction or pathway enrichment analysis. In TCGA, the drugs related CSL or CSV interactions have impact on patient prognosis by deregulating genome, epigenome, tumor microenvironment and genomic stability. Furthermore, in patients with mutations of a CR set (*EP300*, *MSH6*, *NSD2* and *TRRAP*), COAD patients with *MAP2* low expression showed better prognosis and could benefit more from Paclitaxel and Docetaxel.

Mutations of CRs have synthetic viable or lethal effect with other genes by deregulating methylation and gene expression, which were validated by co-expression and co-methylation analysis. Notably, majority of CRs genetic interactions showed positive correlation at methylation level, either in CSL or CSV interaction. Positive correlation at methylation level may be a potential signature of CRs genetic interaction involved in cancer cells.

In the present work, we provided a novel perspective on the identification of personalized biomarkers for drug response and expand the scope of precision oncology via CRs genetic interactions. For instance, *SMARCA4*, a subunit of the SWI/SNF chromatin remodeling complex, and *FLT1 (VEGFR1)* were identified as a CSL interaction in ovarian cancer cells. Most of the Small Cell Carcinoma of the Ovary Hypercalcemic Type (SCCOHT) patients carried variants in *SMARCA4* [36]. *SMARCA4* mutation was associated with sensitivity to four *FLT1 (VEGFR1)* inhibitors in ovarian cancer cells, including axitinib, foretinib, pazopanib and sorafenib (Additional file 1: Fig. S11). Above results suggest SCCOHT patients with mutations of *SMARCA4* may benefit from *FLT1 (VEGFR1)* inhibitors. In addition, *RB1* mutations combined with *CDK4/6* deficiency had synthetic viable effect in lung cancer cells, which indicates that the lung cancer patients with *RB1* mutations could not be suggested to use Palbociclib (Additional file 1: Fig. S1). Experimental verification researches are warranted to elucidate the mechanism of drug sensitivity and resistance, which will be the major focus in our future studies.

Basic drug response was highly heterogeneous among tissue-specific cell lines [37]. Consequently, the drug response analysis in this study was performed on tissue specific cancer cells. However, the amount of specific cancer cell lines was small. Thus, we did not use *FDR* to identify the biomarkers with the limitation of statistical power restricted to the sample size. Nevertheless, drug response-related CSL and CSV interactions were identified by different functional screens and pharmacological datasets. We filtered drug response-related CRs genetic interaction by a combined *P* value via Fisher’s combined probability test. In addition, majority of the genetic interactions have directly or indirectly PPI (Fig. 3A and B) and genomic characterization (Fig. 4A–F) in tissue specific cancers. The aforementioned results indicate that CSL and CSV genetic biomarkers are robust and reliable.

Protein dysregulation in cell lines is valuable and vital data resources to better understand and interpret the CRs genetic interactions. We focused the correlation between some corresponding protein expression of mutation genes and the target genes dependency in lung cancer cell lines due to the limitation of sample numbers, including *PCDH19-ZHX2*, *CHD7-YES1* (Additional file 1: Fig. S12). Furthermore, we speculated that mutation genes form synthetic lethal/viable effects with the target genes via the dysregulation of the corresponding protein.

Furthermore, we found that missense mutations confer to the most effect of both CSL and CSV interactions in the functional screens datasets (shRNA, CRISPR1, CRISPR2), and the second mutation type is nonsense mutation (Additional file 1: Fig. S13 and S14). Further detailed researches are warranted to unravel the mechanism of missense mutations confer to drug sensitivity and resistance in our future studies.

The mutation frequencies of CRs have high heterogeneity in various cancers. In the present work, we focused on single synthetic effects and several united effects related to CRs. A large number of high-order genetic interaction and drug combinations analysis warrant our future work, especially for the CRs belonging to same categories or sharing common functions.

## Conclusions

Overall, we proposed an algorithm to identify CRs genetic interaction and systematically investigated the drug response effect due to the CSL and CSV interaction. By mining drug response-related CR genetic interactions in TCGA, we further discovered their special effect in transcriptional control, epigenetic changes, genomic instability, tumor microenvironment and survival outcome. Regardless, we believe that the biomarkers identified by our work will conductive to predict the mechanism of drug response in cancer treatment and will guide precise targeting of clinical application.

## Supplementary Information

**Additional file 1:** **Fig. S1.** Lung cancer cell lines with *RB1* mutations are resistant to *CDK4/6* inhibitor, **Fig. S2.** Sensitive biomarkers interacted with *CHEK1* in large intestine cancer cell lines, **Fig. S3.** Resistant biomarkers interacted with *CHEK1* in lung cancer cell lines, **Fig. S4.** Functional analysis of the CRs genetic interaction, **Fig. S5.** Differential chromatin accessibility for CSL interactions, **Fig. S6.** Differential chromatin accessibility for CSV interactions, **Fig. S7.** *TUBA1C* subnetwork in drug related CSL network, **Fig. S8.** CRs genetic interactions were related to the prognosis of patients, **Fig. S9.** *MAP2* CSL module mediate poor prognosis in COAD, **Fig. S10.** Vorinostat CSV module induces multiple omics deregulation in COAD, **Fig. S11.** *SMARCA4* mutation was *sensitive* to FLT1 inhibitor in ovarian cancer cell lines, **Fig. S12.** The correlation between protein expression of mutated genes and the target genes dependency in lung cancer cell lines, **Fig. S13.** Comparison of the multi-mutation types conferred to CSL in the functional screen datasets, **Fig. S14**. Comparison of the multi-mutation types conferred to CSV in the functional screens datasets. **Table S1.** Functional screen datasets, **Table S2.** Pharmacogenomics datasets.

**Additional file 2: Table S3.** CRs genetic interactions related to drug response in cancers.

## Data Availability

All data analyzed during this study was publicly available and was provided in the Supplementary Information files. The following supporting information can be downloaded.

## Abbreviations

ATAC-seq: Assay for Transposase-accessible chromatin with sequencing
ATC: Anatomical Therapeutic Chemical Classification System
AUC: The area-under-concentration-response curve
BP: Biological processes
BRCA: Breast cancer
COAD: Colon cancer
CC: Cellular component
CCLE: The Cancer Cell Line Encyclopedia
CGP: Cancer Genome Project
Cr: Chromatin remodeling
CRs: Chromatin regulators
CSL: CRs synthetic lethality
CSV: CRs synthetic viability
CTRP: Cancer Therapeutics Response Portal
Dm: DNA methylation
dMMR: MMR deficient
E: Eraser
EGFR: Epidermal growth factor receptor
GBM: Glioblastoma
GDSC: Genomics of Drug Sensitivity in Cancer
GO: Gene Ontology
HDAC: Histone deacetylases
Hm: Histone modification
HR: Homologous recombination repair
HRD: Homologous recombination deficiency
IC50: The half maximal inhibitory concentration
KEGG: Kyoto Encyclopedia of Genes and Genomes
LGG: Low-grade glioma
LUAD: Lung adenocarcinoma
LUSC: Lung squamous cell carcinoma
MF: Molecular function
MMR: Mismatch repair
MSI-H: Microsatellite instability high
PPI: Protein-protein interaction
R: Reader
SCCOHT: The Small Cell Carcinoma of the Ovary Hypercalcemic Type
shRNA: Short hairSpin RNA
TCGA: The Cancer Genome Atlas
UCEC: Uterine corpus endometrial carcinoma
W: Writer
