# Supplementary material for: A genetic map of the chromatin regulators to drug response in cancer cells
Source: J Transl Med. 2022 Sep 30;20:438. doi: 10.1186/s12967-022-03651-w (PMC9523919; doi:10.1186/s12967-022-03651-w)
Supplement: Supplementary file 1 — Additional file 1: Fig. S1. Lung cancer cell lines with RB1 mutations are resistant to CDK4/6 inhibitor, Fig. S2. Sensitive biomarkers interacted with CHEK1 in large intestine cancer cell lines, Fig. S3. Resistant biomarkers interacted with CHEK1 in lung cancer cell lines, Fig. S4. Functional analysis of the CRs genetic interaction, Fig. S5. Differential chromatin accessibility for CSL interactions, Fig. S6. Differential chromatin accessibility for CSV interactions, Fig. S7. TUBA1C subnetwork in drug related CSL network, Fig. S8. CRs genetic interactions were related to the prognosis of patients, Fig. S9. MAP2 CSL module mediate poor prognosis in COAD, Fig. S10. Vorinostat CSV module induces multiple omics deregulation in COAD, Fig. S11. SMARCA4 mutation was sensitive to FLT1 inhibitor in ovarian cancer cell lines, Fig. S12. The correlation between protein expression of mutated genes and the target genes dependency in lung cancer cell lines, Fig. S13. Comparison of the multi-mutation types conferred to CSL in the functional screen datasets, Fig. S14. Comparison of the multi-mutation types conferred to CSV in the functional screens datasets. Table S1. Functional screen datasets, Table S2. Pharmacogenomics datasets. [file 12967_2022_3651_MOESM1_ESM.pdf]

Additional file 1 for

A Genetic Map of the Chromatin Regulators to Drug Response in Cancer Cells

Additional file 1: Supplementary Figures

Fig. S1. Lung cancer cell lines with *RB1* mutations are resistant to *CDK4/6* inhibitor

Fig. S2. Sensitive biomarkers interacted with *CHEK1* in large intestine cancer cell lines

Fig. S3. Resistant biomarkers interacted with *CHEK1* in lung cancer cell lines

Fig. S4. Functional analysis of the CRs genetic interaction

Fig. S5. Differential chromatin accessibility for CSL interactions

Fig. S6. Differential chromatin accessibility for CSV interactions

Fig. S7.*TUBA1C* subnetwork in drug related CSL network

Fig. S8. CRs genetic interactions were related to the prognosis of patients

Fig. S9. *MAP2* CSL module mediate poor prognosis in COAD

Fig. S10. Vorinostat CSV module induces multiple omics deregulation in COAD

Fig. S11. *SMARCA4* mutation was sensitive to *FLT1* inhibitor in ovarian cancer cell lines

Fig. S12. The correlation between protein expression of mutated genes and the target genes dependency in lung cancer cell lines

Fig. S13. Comparison of the multi-mutation types conferred to CSL in the functional screen datasets

Fig. S14. Comparison of the multi-mutation types conferred to CSV in the functional screens datasets.

Additional file 1: Supplementary Tables

Table S1. Functional screen datasets

Table S2. Pharmacogenomics datasets

Additional file 1: Supplementary Figures

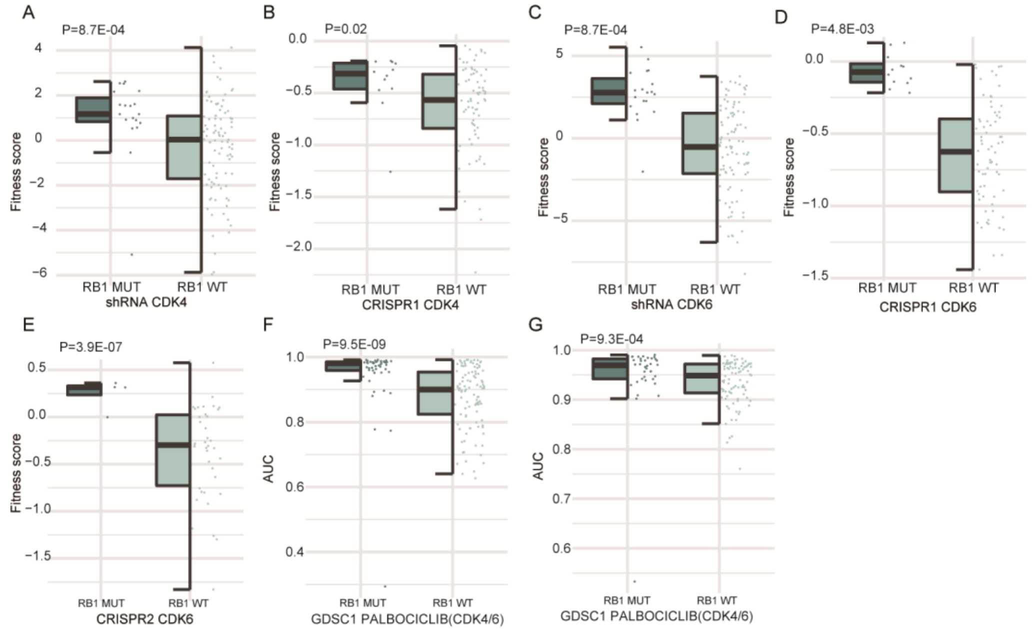

Fig. S1. Lung cancer cell lines with *RB1* mutations are resistant to *CDK4/6* inhibitor

Lung cancer cell lines with *RB1* mutations have better viability when *CDK4* were knocked down in shRNA (A) and CRISPR1 (B). Lung cancer cell lines with *RB1* mutations have better viability when *CDK6* were knocked down in shRNA (C), CRISPR1 (D), CRISPR2 (E). Lung cancer cell lines with *RB1* mutations were resistant to inhibitor of *CDK4/6* in GDSC1 (F) and GDSC2 (G).

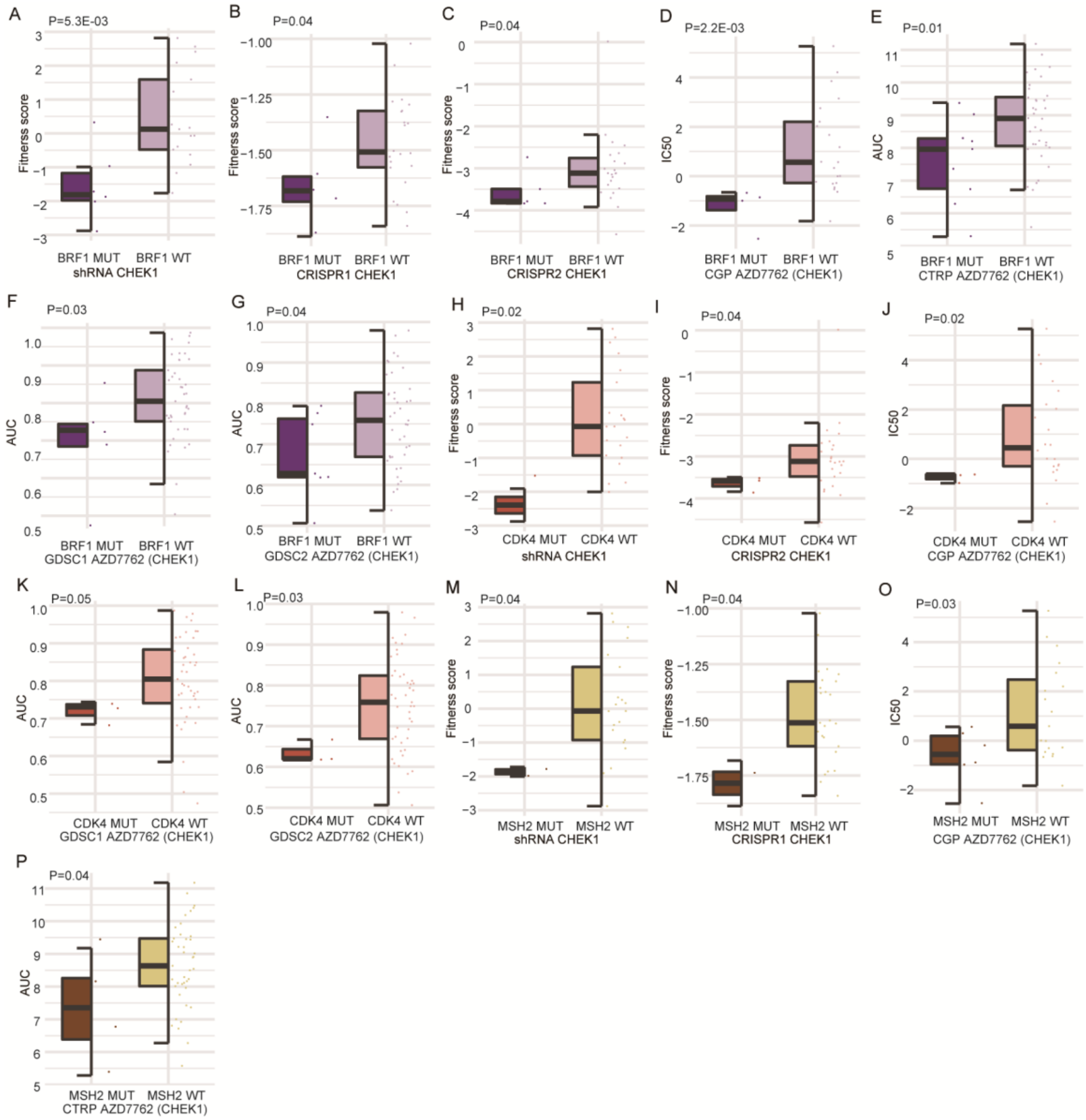

Fig. S2. Sensitive biomarkers interacted with *CHEK1* in large intestine cancer cell lines

Large intestine cancer cell lines with *BRF1* mutations had worse viability when *CHEK1* was knocked down in shRNA (A), CRISPR1 (B) and CRISPR2 (C). Large intestine cancer cell lines with *BRF1* mutations were sensitive to AZD7762 in CGP (D), CTRP (E), GDSC1 (F) and GDSC2 (G). Large intestine cancer cell lines with *CDK4* mutations had worse viability when *CHEK1* is knocked down in shRNA (H) and CRISPR2 (I). Large intestine cancer cell lines with *CDK4* mutation were sensitive to AZD7762 in CGP (J), GDSC1 (K) and GDSC2 (L). Large intestine cancer cell lines with *MSH2* mutations had worse viability when *CHEK1* was knocked down in

shRNA (M) and CRISPR1 (N). Large intestine cancer cell lines with *MSH2* mutation were sensitive to AZD7762 in CGP (O) and CTRP (P).

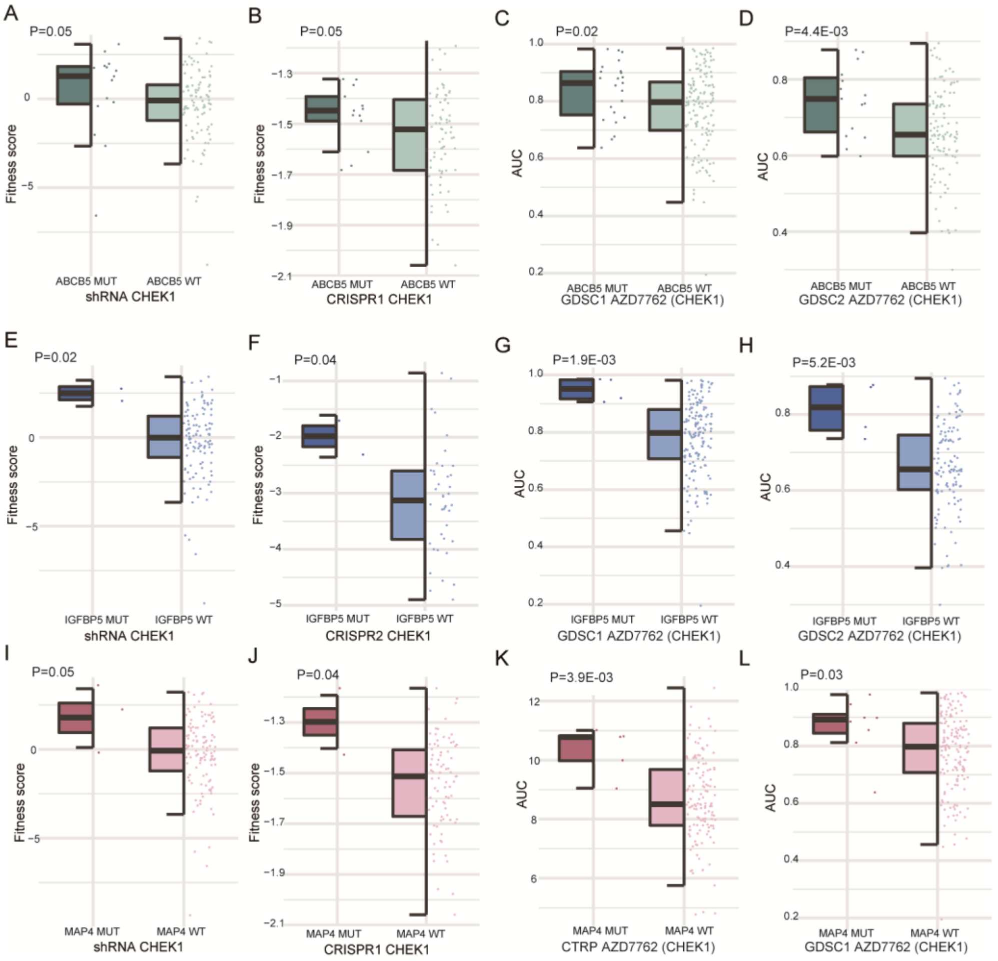

Fig. S3. Resistant biomarkers interacted with *CHEK1* in lung cancer cell lines

Lung cancer cell lines with *ABCB5* mutations had better viability when *CHEK1* was knocked down in shRNA (A) and CRISPR1 (B). Lung cell lines with *ABCB5* mutation were resistance to AZD7762 in GDSC1 (C) and GDSC2 (D). Lung cancer cell lines with *IGFBP5* mutation had better viability when *CHEK1* was knocked down in shRNA (E) and CRISPR2 (F). Lung cancer cell lines with *IGFBP5* mutations were resistant to AZD7762 in GDSC1 (G) and GDSC2 (H). Lung cancer cell lines with *MAP4* mutations had better viability when *CHEK1* was knocked down in shRNA (I) and CRISPR1 (J). Lung cancer cell lines with *MAP4* mutations were resistant to

AZD7762 in CTRP (K) GDSC1 (L).

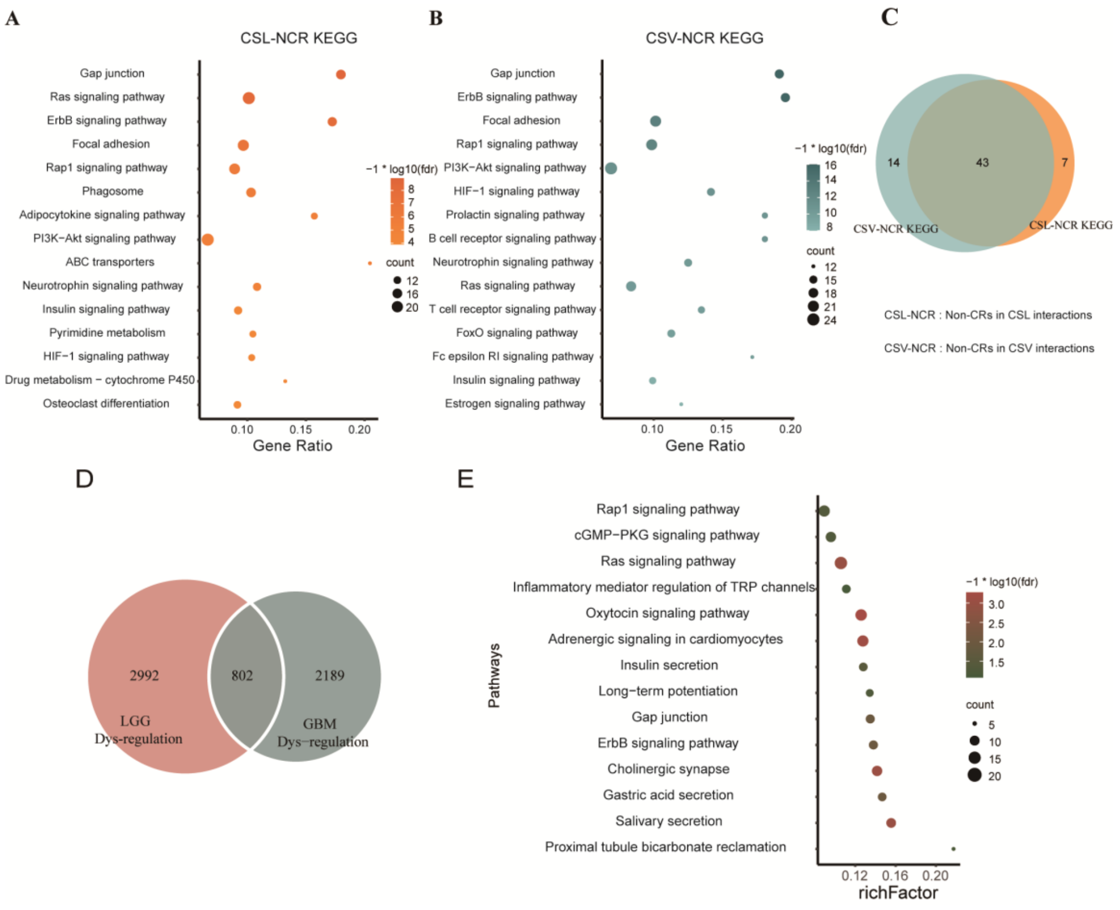

Fig. S4. Functional analysis of the CRs genetic interaction

Genes interacted with CRs in CSL (A) and CSV (B) genetic interactions were enriched with KEGG pathways. Only top 15 significantly enriched pathways were showed. (C) Overlapping of pathways enriched in CSL and CSV. (D) The overlapping of differential expression genes between GBM and LGG patients with *TP53* mutations, according to the median expression value of *KIT*. (E) The overlapping genes in (C) enriched in KEGG pathways.

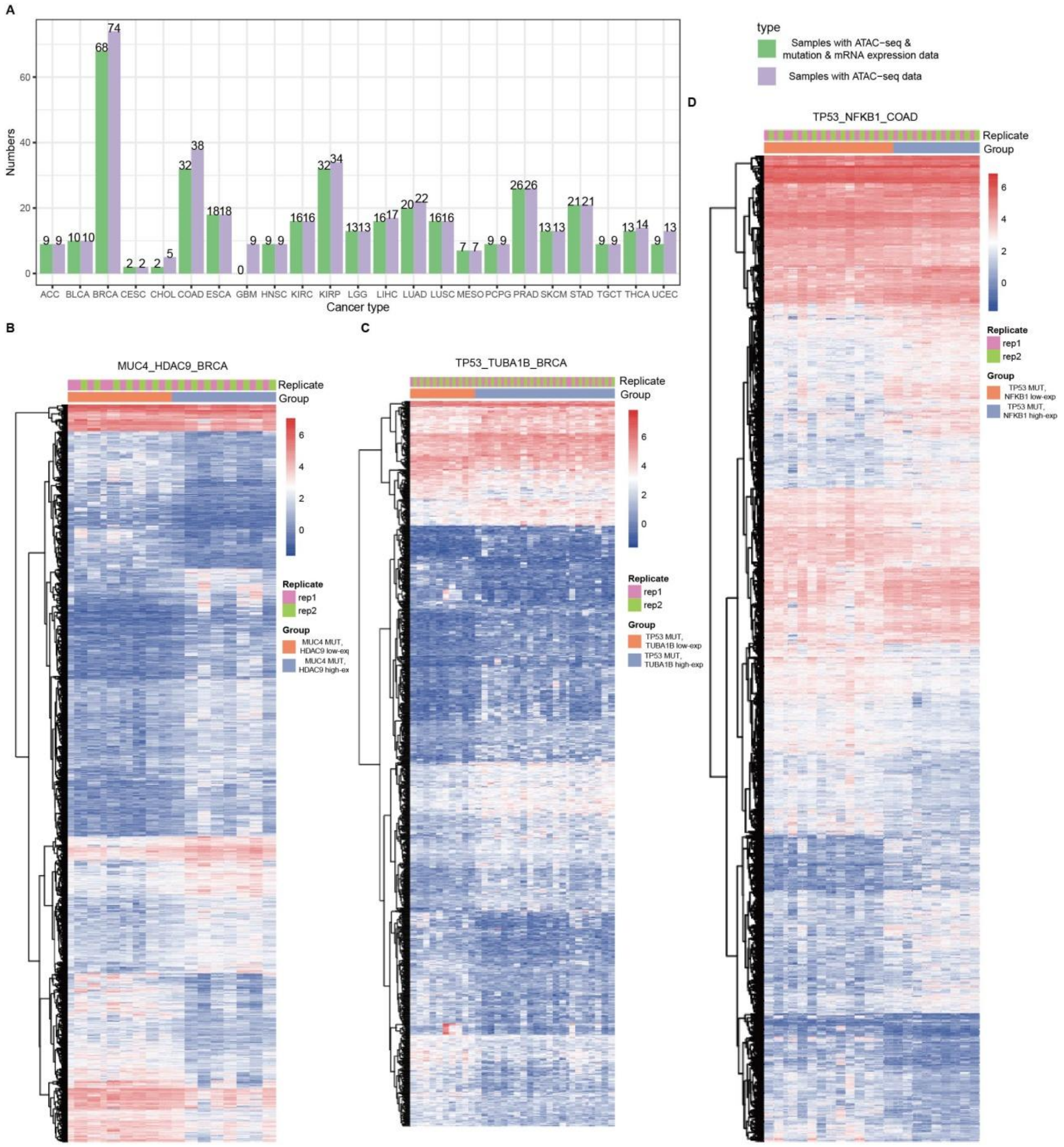

Fig. S5. Differential chromatin accessibility for CSL interactions. (A) The sample size of patients with multiple omics datasets in TCGA. Hierarchical clustering of the differentially accessible peaks between patients with and without *MUC4-HDAC9* interaction (B), *TP53-TUBA1B* interaction (C) in breast cancer and *TP53-NFKB1* interaction （D） in colon cancer, respectively. Colors represent peak count data.

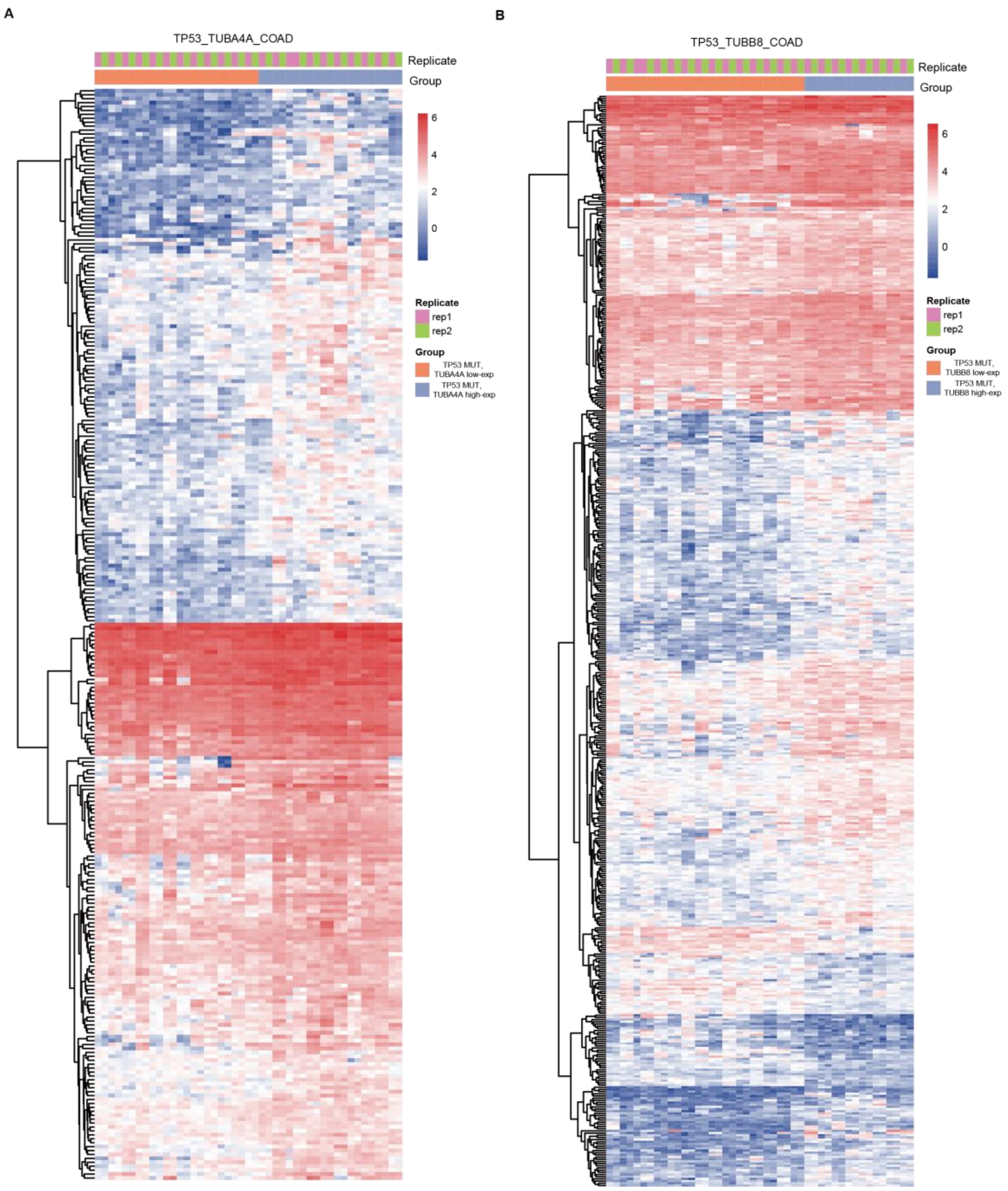

Fig. S6. Differential chromatin accessibility for CSV interactions. Hierarchical clustering of the differentially accessible peaks between colon cancer patients with and without *TP53*-*TUBA4A* interaction (A) and *TP53*-*TUBB8* interaction (B). Colors represent peak count data.

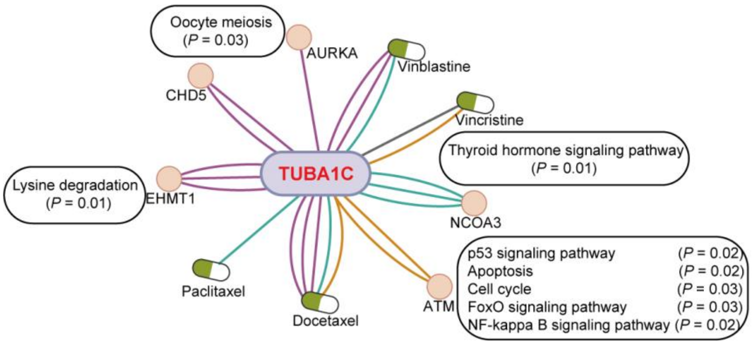

Fig. S7. *TUBA1C* subnetwork in drug related CSL network

TUBA1C subnetwork and functional analysis of the genes interacted with *TUBA1C* in drug related CSL network.

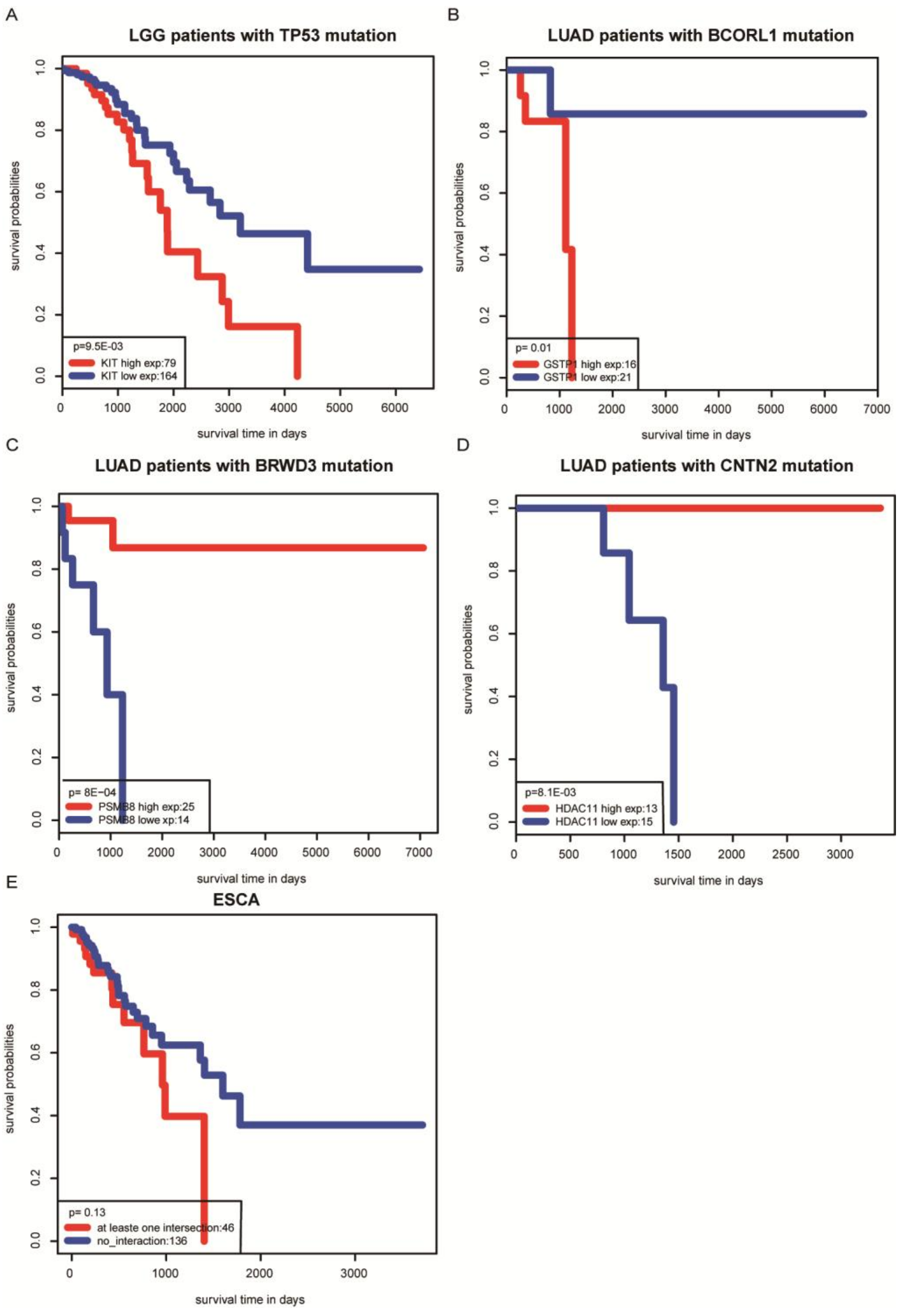

Fig. S8. CRs genetic interactions were related to the prognosis of patients.

(A) The Kaplan-Meier overall survival of *TP53* mutation carriers in LGG patients in two groups as follows: *KIT* low-expression and *KIT* high-expression.(B) The Kaplan-Meier overall survival of *BCORL1* mutation carriers in LUAD patients in two groups as follows: *GSTP1* low-expression and *GSTP1* high-expression. (C) The Kaplan-Meier overall survival of *BRWD3* mutation carriers

in LUAD patients in two groups as follows: *PSMB8* low-expression and *PSMB8* high-expression. (D) The Kaplan-Meier overall survival of *CNTN2* mutation carriers in LUAD patients in two groups as follows: *HDAC11* low-expression and *HDAC11* high-expression. (E) The Kaplan-Meier overall survival of ESCA patients in two groups as follows: at least one CSV interaction and without CSV interactions.

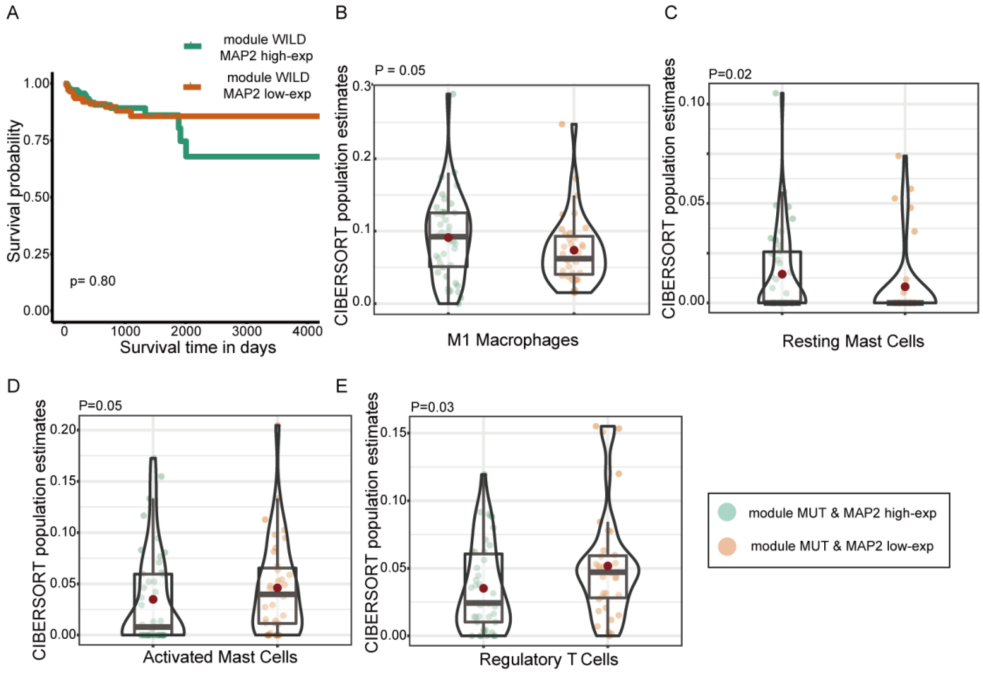

Fig.S9. ***MAP2*** **CSL module mediate poor prognosis in COAD**

(A) The Kaplan-Meier survival analysis of classifications generated by *MAP2* expression in the patients without module partner genes mutation. The distribution of M1 macrophages (B), Resting Mast cells (C), Activated Mast cells (D) and Tregs (E) infiltration for 2 subtypes with *MAP2* high or low expression in COAD patients carrying mutations of *MAP2* CSL module. *P* values were calculated by one-sided Wilcoxon rank-sum test.

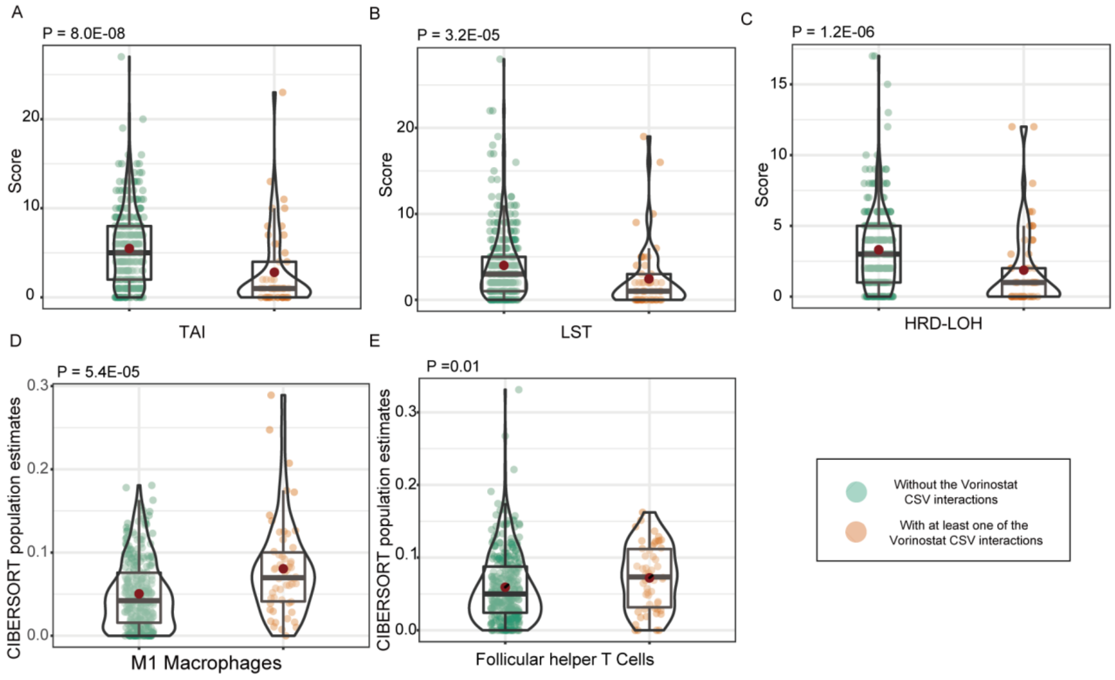

Fig.S10. Vorinostat CSV module induces multiple omics deregulation in COAD

The comparison of HRD-telomeric allelic imbalance score (A), HRD-large-scale state transition score (B), HRD-loss of heterozygosity score (C), M1 macrophages (D) and follicular helper T cells (E) infiltration for 2 subtypes. *P* values were calculated by one-sided Wilcoxon rank-sum test.

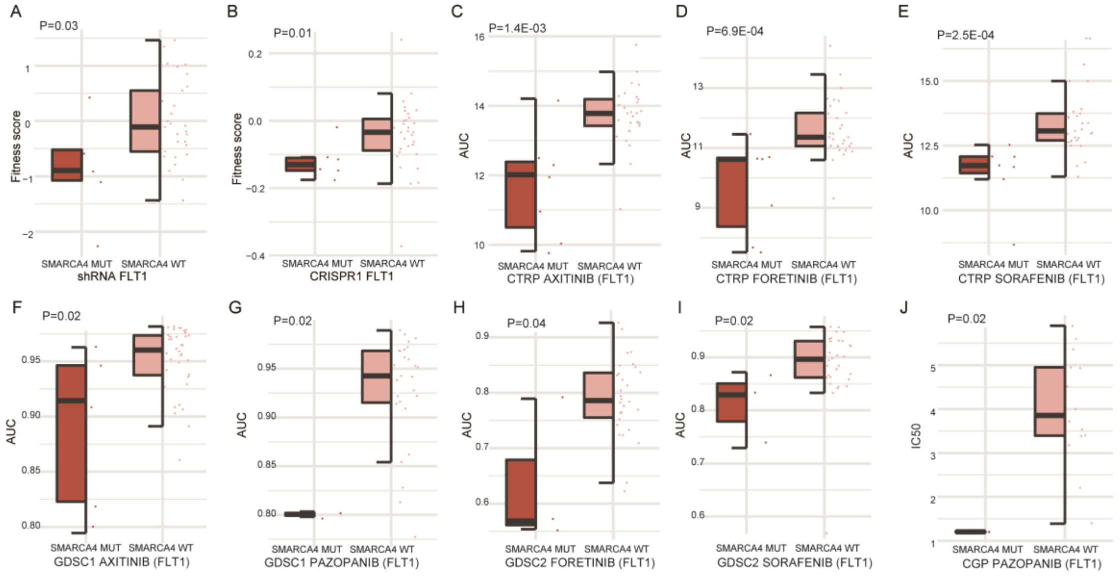

Fig. S11. *SMARCA4* mutation was sensitive to *FLT1* inhibitor in ovarian cancer cell lines.

Ovarian cancer cell lines with *SMARCA4* mutation had worse viability when *FLT1* were knocked down in shRNA (A) and CRISPR1 (B). (C)-(J).Ovarian cancer cell lines with *SMARCA4* mutation were sensitive to inhibitor of *FLT1* in CTRP,GDSC1,GDSC2.

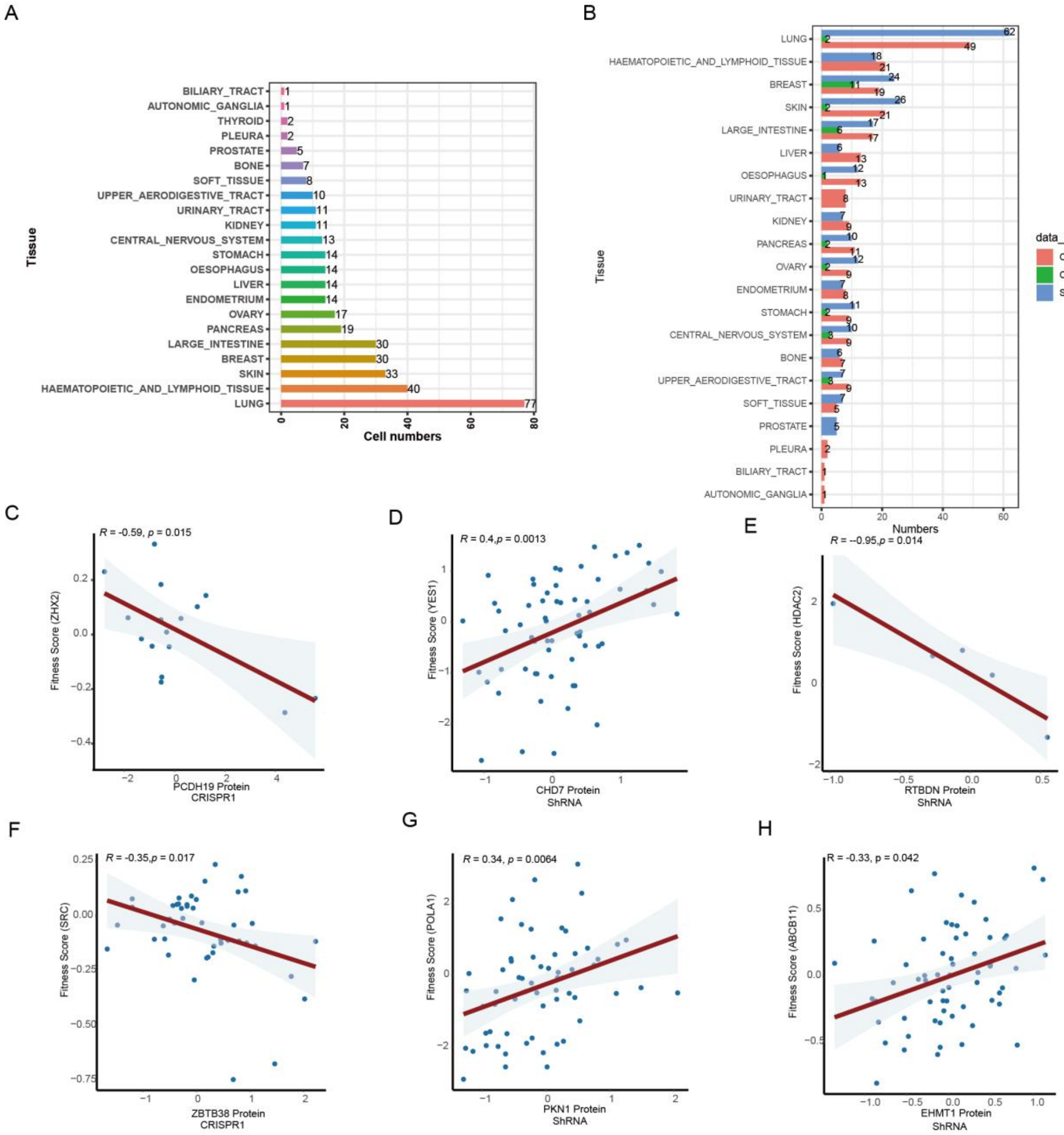

Fig. S12. The correlation between protein expression of mutated genes and the target genes dependency in lung cancer cell lines. (A). Overview of the cell line numbers detected by mass spectrometry. (B). Overview of the intersection cell line numbers detected by mass spectrometry and CRISPR/shRNA screens. Examples of CSL *PCDH19-ZHX2*(C), *CHD7-YES1*(D), *RTBDN-HDAC2* (E) and CSV *ZBTB38-SRC*(F), *PKN1-POLA1*(G), *EHMT1-ABCB11*(H)

interactions showed correlations between corresponding protein expression of mutated genes and the target genes dependency score in lung cancer cell lines.

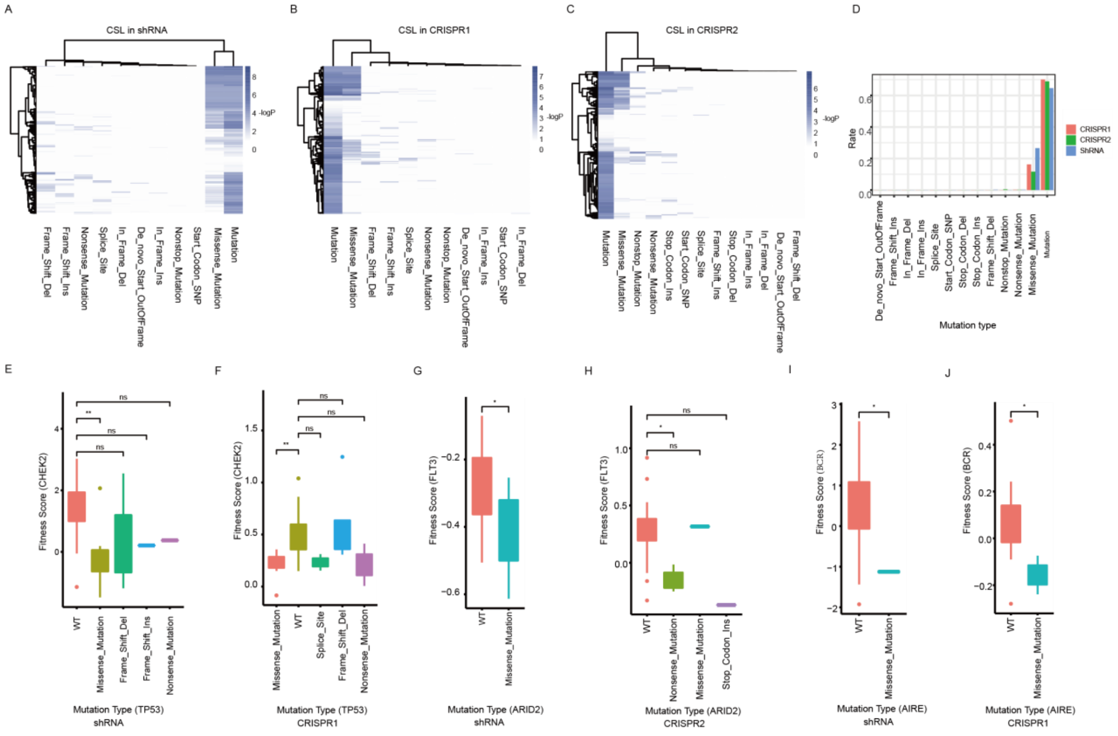

Fig. S13. Comparison of the multi-mutation types conferred to CSL in the functional screen datasets. Cell lines with different partner gene mutation types result in CSL effect in shRNA (A), CRISPR1 (B), and CRISPR1 (C). Heatmap of different mutation types show the *–logP*. *P* values were calculated by one-sided Wilcoxon rank-sum test when comparison the target gene fitness score of cell lines with partner gene wild-type and specific type mutation. (D).The ratio of specific mutation type conferred to synthetic lethality effect in the functional screen datasets. (E-J).The comparison of target gene fitness scores with distinct mutation types. * indicates $P < 0.05$, ** indicates $P < 0.005$.

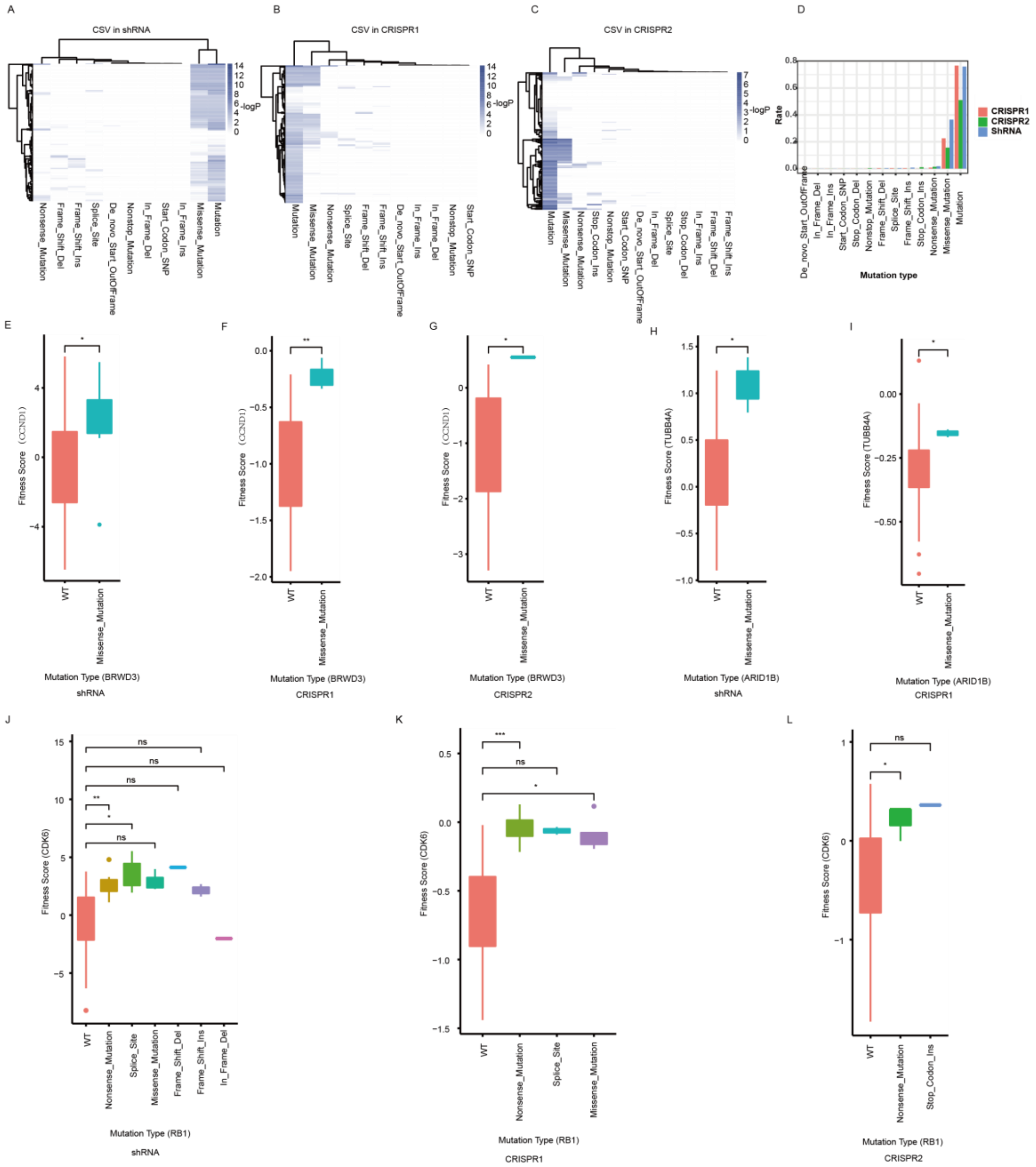

Fig. S14. Comparison of the multi-mutation types conferred to CSV in the functional screens datasets. Cell lines with different partner gene mutation types result in CSV effect in shRNA (A), CRISPR1 (B) and CRISPR1 (C). Heatmap of the different mutation types shows the *–logP*. *P* values were calculated by one-sided Wilcoxon rank-sum test when comparison the target gene fitness score of cell lines with partner gene wild-type and specific type mutation. (D).The ratio of specific mutation type conferred to synthetic viability effect in the functional screen datasets. (E-L).The comparison of target gene fitness scores with distinct mutation types. * indicates *P* <

0.05, ** indicates $P < 0.005$, *** indicates $P < 0.0005$.

Additional file 1: Supplementary Tables

Table S1. Functional screen datasets

Datasets	Cell lines size	Cell line sources	Data Sources
CRISPR1	625	CCLE	https://depmap.org/portal/
CRISPR2	325	COSMIC	https://score.depmap.sanger.ac.uk/
shRNA	501	CCLE	https://depmap.org/portal/

Table S2. Pharmacogenomics datasets

Datasets	Cell line sources	Sentivity measure	Cell lines size	Drug size	Data Sources
CCLE	CCLE	IC50	504	24	https://portals.broadinstitute.org/ccle
CGP	COSMIC	IC50	639	130	http://cancer.sanger.ac.uk/cosmic
CTRP	CCLE	AUC	835	481	https://www.broadinstitute.org/ctrp/
GDSC1	COSMIC	AUC	988	304	https://www.cancerrxgene.org/
GDSC2	COSMIC	AUC	811	169	https://www.cancerrxgene.org/
